# Characterization of the Avian Trojan Gene Family Reveals Contrasting Evolutionary Constraints

**DOI:** 10.1371/journal.pone.0121672

**Published:** 2015-03-24

**Authors:** Petar Petrov, Riikka Syrjänen, Jacqueline Smith, Maria Weronika Gutowska, Tatsuya Uchida, Olli Vainio, David W Burt

**Affiliations:** 1 Institute of Diagnostics, Department of Medical Microbiology and Immunology, University of Oulu, Oulu, Finland; 2 Nordlab Oulu, Oulu University Hospital, Oulu, Finland; 3 Medical Research Center Oulu, Oulu University Hospital and University of Oulu, Oulu, Finland; 4 Division of Genetics and Genomics, The Roslin Institute and R(D)SVS, University of Edinburgh, Roslin, United Kingdom; Laboratoire de Biologie du Développement de Villefranche-sur-Mer, FRANCE

## Abstract

“Trojan” is a leukocyte-specific, cell surface protein originally identified in the chicken. Its molecular function has been hypothesized to be related to anti-apoptosis and the proliferation of immune cells. The Trojan gene has been localized onto the Z sex chromosome. The adjacent two genes also show significant homology to Trojan, suggesting the existence of a novel gene/protein family. Here, we characterize this Trojan family, identify homologues in other species and predict evolutionary constraints on these genes. The two Trojan-related proteins in chicken were predicted as a receptor-type tyrosine phosphatase and a transmembrane protein, bearing a cytoplasmic immuno-receptor tyrosine-based activation motif. We identified the Trojan gene family in ten other bird species and found related genes in three reptiles and a fish species. The phylogenetic analysis of the homologues revealed a gradual diversification among the family members. Evolutionary analyzes of the avian genes predicted that the extracellular regions of the proteins have been subjected to positive selection. Such selection was possibly a response to evolving interacting partners or to pathogen challenges. We also observed an almost complete lack of intracellular positively selected sites, suggesting a conserved signaling mechanism of the molecules. Therefore, the contrasting patterns of selection likely correlate with the interaction and signaling potential of the molecules.

## Introduction

The immune system protects an individual from pathogens in the surrounding environment. Driven by a constant need to adapt to novel pathogen challenges, genes of the immune system are often forced to evolve faster compared to other genes [1]. As a result, a number of immune system genes and the proteins they encode display variability that is currently among the highest known in animal species [2].

In jawed vertebrates, the immune system can be divided into innate, which presents the first line of host defense and adaptive, which provides a more sophisticated means of fighting pathogens [3,4]. Positive Darwinian selection has been described for a variety of genes associated with adaptive immunity, among which are the major histocompatibility complex (MHC) molecules [5–7], the immunoglobulin heavy chain (IgH) [8] and the common leukocyte antigen, CD45 [9]. Also, signatures of positive selection have been shown for genes associated with innate immunity, like the Toll-like receptor (TLR) 1 family [10], TLR4 and TLR7 [11]. Evidence of positive selection has been found for other genes, associated with both innate and adaptive immunity, such as chemokine receptors [12], interleukins (IL) and IL receptors [13,14]. However, in other instances, genes related to host defense have been shown to be highly conserved. This is likely a result of negative purifying selection acting to eliminate deleterious mutations in molecules that need to stay unchanged. Such is the case of C-C chemokine receptor 5 (CCR5), which has a conserved conformation [15] and the common gamma chain (γc) of IL receptors [14], which acts as a hub protein to other associating receptor chains.

The molecular tools of host defense involve a variety of proteins many of which are already known, while others are yet to be discovered. Aiming to identify novel proteins related to the immune system, we cloned a previously unknown chicken (*Gallus gallus*) protein from an embryonic day 13 (E13) thymus cDNA library. The molecule is a leukocyte-specific, cell surface protein that we named "Trojan" and characterized previously [16]. The tissue distribution of its transcript closely follows that of CD45, while the protein is found on the surface of lymphocyte subpopulations and macrophages. Based on our detailed analysis of developing thymocytes, we hypothesized an anti-apoptotic and/or proliferative function for Trojan.

The cloned Trojan cDNA is about 2.1 Kb, with coding DNA sequence (CDS) of about 1.5 Kb. It translates to a 494 amino acids long, type I transmembrane protein that is likely to be glycosylated. The extracellular part of Trojan is predicted to have a signal peptide, followed by a complement control protein (CCP) domain and a pair of fibronectin type III (FN3) domains. The cytoplasmic tail of Trojan is short and has a region of four positively charged amino acids and two putative serine phosphorylation sites.

As described previously, Trojan nucleotide sequence was mapped to the Z sex chromosome of the chicken genome [16]. However, we also found the gene on BAC clone CH261–99K12, which contained the complete and uninterrupted Trojan sequence. The neighboring upstream and downstream genes were shown to be highly homologous to Trojan and we therefore suggested the existence of a novel Trojan gene/protein family.

In this paper, we present a detailed analysis of the Trojan gene family in chicken and other avian and non-avian species. The recent advances in the chicken genome assembly and gene annotations (Galgal4), allowed us to identify the proteins coded by the two Trojan-related genes. With the rapid accumulation of genomic sequence data from numerous species, we also found homologous sequences in ten other avian species, as well as three reptiles and West Indian ocean coalecanth fish. Since the majority of these genome assemblies were in the form of separate scaffolds, we performed manual scaffold assembly and gene modeling. Obtaining the predicted Trojan-like genes allowed us to perform phylogenetic analyzes of the family members and determine the pattern of evolutionary selection they have been subjected to. We found strong evidence of positive Darwinian selection mostly in the extracellular domain, while other parts of the proteins appeared to be under purifying negative selection. These contrasting evolutionary patterns likely correlate with the role of the protein domains and cytoplasmic tails.

## Results

### Trojan gene family in chicken

**Fig 1:**
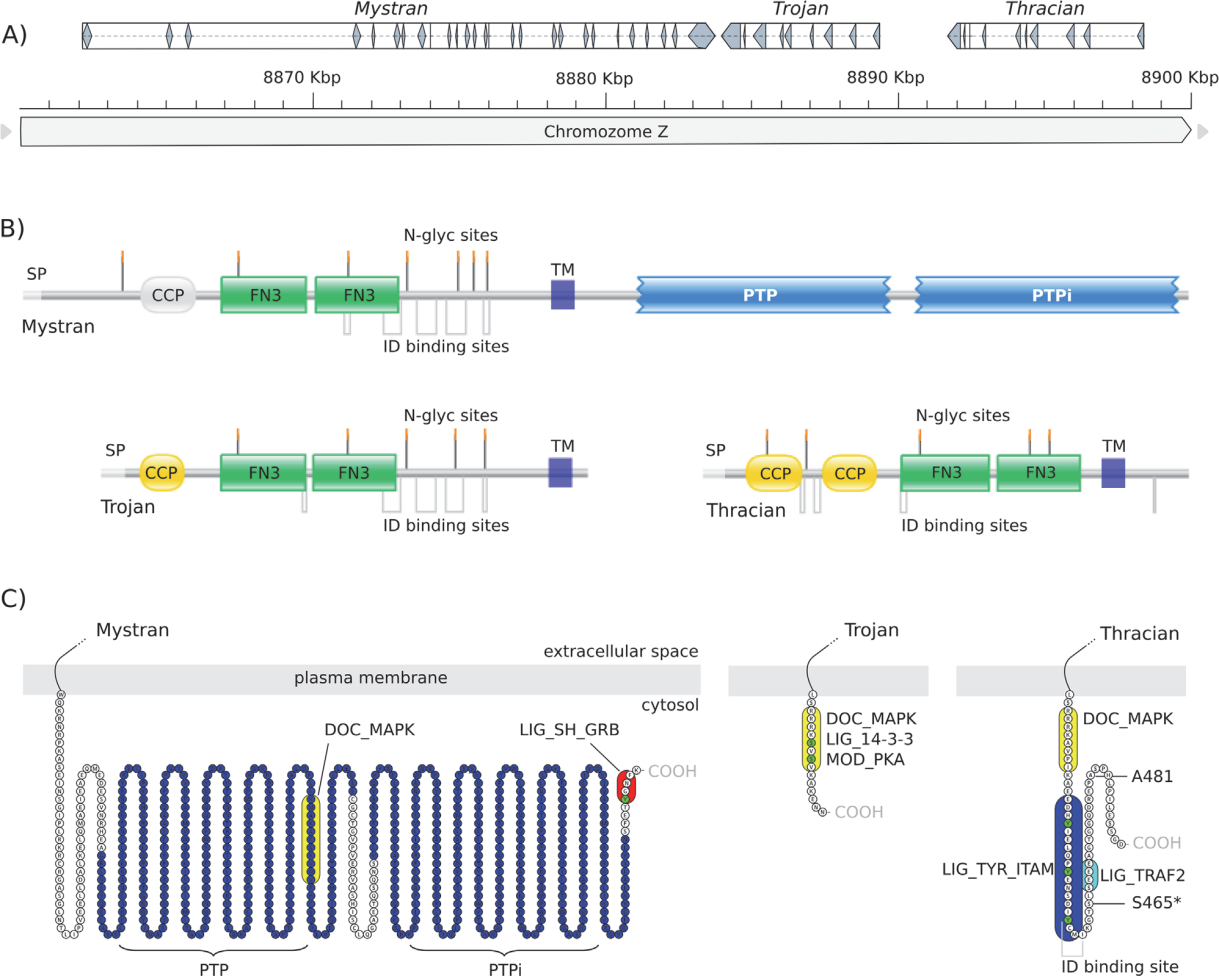
The Trojan family in chicken. A) *Mystran*, *Trojan* and *Thracian* on chicken chromosome Z. Genes are represented as hollow boxes showing their direction. Exons are shown as filled fragments within the gene boxes; B) The overall topology organization of the Trojan family proteins. Complement control protein (CCP) domains, fibronectin type III domains (FN3) and protein tyrosine phosphatase domains (PTP) are labeled. Signal peptides (SP), domains, transmembrane regions (TM), N-glycosylation (N-glyc) sites and intrinsically disordered (ID) binding sites are indicated. The Mystran CCP domain is shown in gray scale, as it was predicted slightly below threshold, but had the expected position. C) The cytoplasmic tails of Mystran, Trojan and Thracian are shown in a “snake” amino acids view. Short functional motifs are indicated: MAPK docking motif (DOC_MAPK), Grb association motif (LIG_SH_GRB), 14-3-3 docking motif (LIG_14-3-3), PKA phosphorylation motif (MOD_PKA), ITAM (LIG_TYR_ITAM) and TRAF2 interacting motif (LIG_TRAF2). For Thracian, the positions of cytoplasmic sites identified to be under positive evolutionary selection with probability higher than 90% and 95% (*) are indicated.

The Trojan gene sequence is found on chicken chromosome Z in the NCBI (National Center for Biotechnology Information) genome database. The neighboring two genes code for putative proteins bearing significant homology to Trojan and to each other (Fig. 1A). In the chicken genome (Galgal4), these are denoted as a receptor type protein tyrosine phosphatase (rPTP), that we named “Mystran” and another uncharacterized transmembrane protein, that we named “Thracian”. The names are simply derived from geographical regions near ancient Troy, to be consistent with the name of Trojan, the gene identified first. The *Mystran* gene is annotated to have 26 exons, stretching over 26,5 Kb of the genomic positive strand sequence. It is followed by the 5,4 Kb gene of *Trojan*, which has 10 exons and resides on the opposite strand. *Thracian* is also found on the negative strand upstream from *Trojan*, has 10 exons and spans a ∼6,7 Kb genomic region. The total genomic range of the family covers about 36 Kb and is bordered by genes *RUSC2* (RUN and SH3 domain containing 2) and *TESK1* (testis-specific kinase 1). Detailed coordinates of the genes and their accession numbers can be found in the Materials and Methods section.

**Fig 2:**
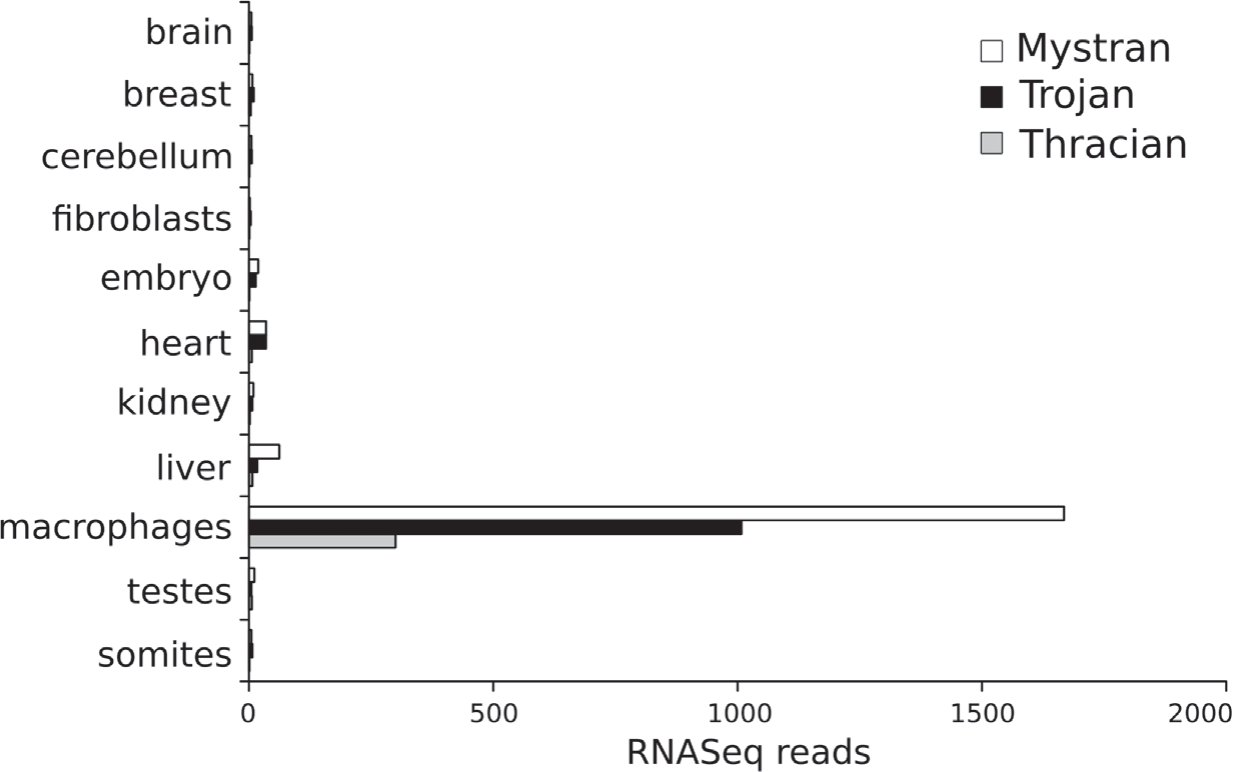
Expression of chicken Mystran, Trojan and Thracian. The relative expression levels of *Mystran*, *Trojan* and *Thracian* genes are presented as RNASeq reads from different organs, tissues, or cell types. Data from Ensembl 75.

Evidence for the expression of the three genes was obtained by *in silico* analyzes using the RNAseq data present in the Ensembl database (Fig. 2), in addition to the tissue distribution of Trojan reported previously [16]. *Mystran*, *Trojan* and *Thracian*, showed varying levels of expression in a number of tissues and cell types, the highest of which appeared to be in macrophages.

Mystran, Trojan and Thracian are suggested to be type I transmembrane proteins that share a similar domain organization in their extracellular parts (Fig. 1B). Pairwise sequence alignments indicated highest similarity between Trojan and Mystran, with an overall identity of 78.4%. Trojan and Thracian had overall identity of 49.5%, while Mystran and Thracian had the lowest overall identity of 44.8%.

Mystran is an 1186 amino acids long protein, predicted to have a CCP domain and two FN3 domains in its glycosylated extracellular part. Its intracellular region has two consecutive PTP domains and two short functional motifs (SFM) (Fig. 1C). One SFM is predicted as a docking site for mitogen-activated protein kinase (MAPK), and the other as a Growth factor receptor-bound protein 2 (Grb2) Src Homology 2 (SH2) domains binding motif.

Trojan is 494 amino acids long, with a glycosylated extracellular region that bears a CCP domain followed by two FN3 domains [16]. Its short cytoplasmic tail has several overlapping SFM (Fig. 1C), suggested as a MAPK docking site, a binding motif for 14-3-3 proteins and a protein kinase A (PKA) phosphorylation site.

Thracian is predicted to be a 493 amino acid long protein, that has pairs of CCP and FN3 domains within its glycosylated extracellular part. The cytoplasmic tail has three SFM (Fig. 1C): an immuno-receptor tyrosine-based activation motif (ITAM) is found between a MAPK docking site and a TNF receptor-associated factors 2 (TRAF2) binding site.

We also predicted intrinsically disordered (ID) region binding sites in all three family members. They were found almost exclusively between, or at the border of the identified domains (Fig. 1B). For Mystran or Trojan, five extracellular ID binding sites were found, while for Thracian, there were three extracellular sites and one intracellular (Fig. 1B, C).

### The Trojan family exists in other avian species

By performing a series of sequence similarity searches, we found genomic regions homologous to chicken *Mystran*, *Trojan* or *Thracian* from ten other avian species (Table 1). These were: *Anas platyrhynchos* (wild duck, mallard), *Corvus brachyrhynchos* (american crow), *Cuculus canorus* (common cuckoo), *Falco peregrinus* (peregrine falcon), *Ficedula albicollis* (collared flycatcher), *Geospiza fortis* (medium ground finch), *Meleagris gallopavo* (wild turkey), *Melopsittacus undulatus* (budgerigar, common parakeet), *Opisthocomus hoazin* (hoatzin, canje pheasant) and *Taeniopygia guttata* (zebra finch). For many species, we found the genes covering more than one scaffold, leaving the sequence split and sometimes incomplete. When required, we manually joined scaffolds using the orientation of the genes in chicken as a reference to direct the assembly. We then used chicken Mystran, Trojan and Thracian to model the gene homologues from these species. Overall, the homologous genes appeared to have the same positions and orientations as in the chicken: *Mystran* followed by *Trojan* and *Thracian* on the opposite strand (S1A Fig.). One exception was *C*. *canorus*, where we found two Trojan genes, described in more detail in the “Phylogenetic analysis” section.

**Table 1:** Trojan family genes in avian and non-avian species.

Gene name	Database ID (NCBI)	Reference Number (NCBI)	Predicted gene coordinates	Orientation
*Avian genes*				
*MYS_ANAPL*	scaffold3198	NW_004679491.1	2101–19824	plus
*TRO_ANAPL*	scaffold3198	NW_004679491.1	25334–21537	minus
*THR_ANAPL*	scaffold3514	NW_004679800.1	7158–15026	plus
*MYS_CORBR*	scaffold139	KK719755.1	24708–6970	minus
*TRO_CORBR →*	scaffold140	KK717827.1	1362390–1363028 →	plus
*TRO_CORBR*	scaffold139	KK719755.1	1–4804	plus
*THR_CORBR*	scaffold140	KK717827.1	1352341–1358982	plus
*MYS_CUCCA*	scaffold483	KL447474.1	1797517–1822404	plus
*TRO1_CUCCA*	scaffold483	KL447474.1	1832626–1824723	minus
*TRO2_CUCCA*	scaffold483	KL447474.1	1847004–1837135	minus
*THR_CUCCA*	scaffold483	KL447474.1	1859639–1850416	minus
*MYS_FALPE →*	C10295565_1	NW_004936052.1	2470–1 →	minus
*MYS_FALPE*	scaffold348_1	NW_004930102.1	31933–21297	minus
*TRO_FALPE*	scaffold348_1	NW_004930102.1	11621–18923	plus
*THR_FALPE*	scaffold348_1	NW_004930102.1	993–5823	plus
*MYS_FICAL*	N00377	NW_004775827.1	44655–14629	minus
*TRO_FICAL*	N00377	NW_004775827.1	4801–11236	plus
*THR_FICAL*	N00129	NW_004775826.1	31992–3152	minus
*MYS_GEOFO →*	C13346903	JH749265.1	2–898 →	plus
*MYS_GEOFO →*	C13853812	JH742003.1	1–3519 →	plus
*MYS_GEOFO*	scaffold4670	JH740780.1	1–6016	plus
*TRO_GEOFO*	scaffold1509	JH740316.1	141695–144904	plus
*THR_GEOFO*	scaffold1509	JH740316.1	130683–139060	plus
*MYS_MELGA*	Chromosome Z	NC_015041.1	9304150–9324725	plus
*MYS_MELUN*	scf900160276923	JH556470.1	57058–37500	minus
*TRO_MELUN*	scf900160276923	JH556470.1	29926–35443	plus
*THR_MELUN →*	scf900160274638	JH554185.1	950–1 →	minus
*THR_MELUN*	scf900160259551	JH539098.1	1243–326	minus
*MYS_OPPHO*	scaffold569	KK736078.1	1320257–1344925	plus
*TRO_OPPHO*	scaffold569	KK736078.1	1354221–1347799	minus
*THR_OPPHO*	scaffold569	KK736078.1	1365487–1356898	minus
*MYS_TAEGU*	Chromosome Z	NC_011493.1	39643887–39669696	plus
*TRO_TAEGU*	Chromosome Z	NC_011493.1	39676959–39672793	minus
*THR_TAEGU*	Chromosome Z	NC_011493.1	39688288–39679555	minus
*Non-avian genes*				
*PP_ANOCA*	chrUn0393	GL343585.1	174373–223856	plus
*TP_ANOCA*	chrUn0393	GL343585.1	434289–412843	minus
*PP_CHEMY*	scaffold1093	KB480077.1	2501–66524	plus
*PP_CHRPI →*	Scfld2946	JH586667.1	14548–23778 →	plus
*PP_CHRPI*	Scfld1664	JH585500.1	1–41787	plus
*TP_CHRPI →*	Scfld6634	JH589385.1	1221–4974 →	plus
*TP_CHRPI*	Scfld1664	JH585500.1	105715–50952	minus
*PP_LATCH*	scaffold00761	JH127322.1	548001–727560	plus
*TP_LATCH*	scaffold00761	JH127322.1	474809–356096	minus

Gene names combine the respective homologue: Mystran (MYS), Trojan (TRO), Thracian (THR), Protein phosphatase (PP) or Transmembrane protein (TP) and the species abbreviation. Avian species: *A*. *platyrhynchos* (ANAPL), *C*. *brachyrhynchos* (CORBR), *C*. *canorus* (CUCCA), *F*. *peregrinus* (FALPE), *F*. *albicollis* (FICAL), *G*. *fortis* (GEOFO), *M*. *gallopavo* (MELGA), *M*. *undulatus* (MELUN), *O*. *hoazin* (OPPHO), *T*. *guttata* (TAEGU); Non-avian species: *A*. *carolinensis* (ANOCA), *C*. *mydas* (CHEMY), *C*. *picta* (CHRPI), *L*. *chalumnae* (LATCH). An arrow indicates a gene found on more than one scaffold and the direction the scaffolds were combined.

We performed further sequence similarity searches and identified gene regions bearing homology to the Trojan family in non-avian species (Table 1). First, we predicted two genes from *Anolis carolinensis* (carolina anole lizard, green anole) that code for a putative protein phosphatase and a transmembrane protein. Using their deduced protein sequences, we modeled homologous genes in two reptilian species—*Chelonia mydas* (green sea turtle), *Chrysemys picta* (painted turtle) and one fish species—*Latimeria chalumnae* (West Indian ocean coalecanth) (S1B Fig.). Our searches did not find homologous sequences in *Xenopus tropicalis* (Western clawed frog) or *Danio rerio* (zebra fish).

Searches against mammalian sequences databases did not return hits with a significant identity score. We also searched the genomes of mammalian species, for any evidence of the Trojan family between genes *RUSC2* and *TESK1*. Among them were *Mus musculus* (mouse) and *Homo sapiens* (human), but no trace of *Mystran*, *Trojan* or *Thracian* genes was found.

### Trojan family proteins have similar topology and share a degree of homology

**Fig 3:**
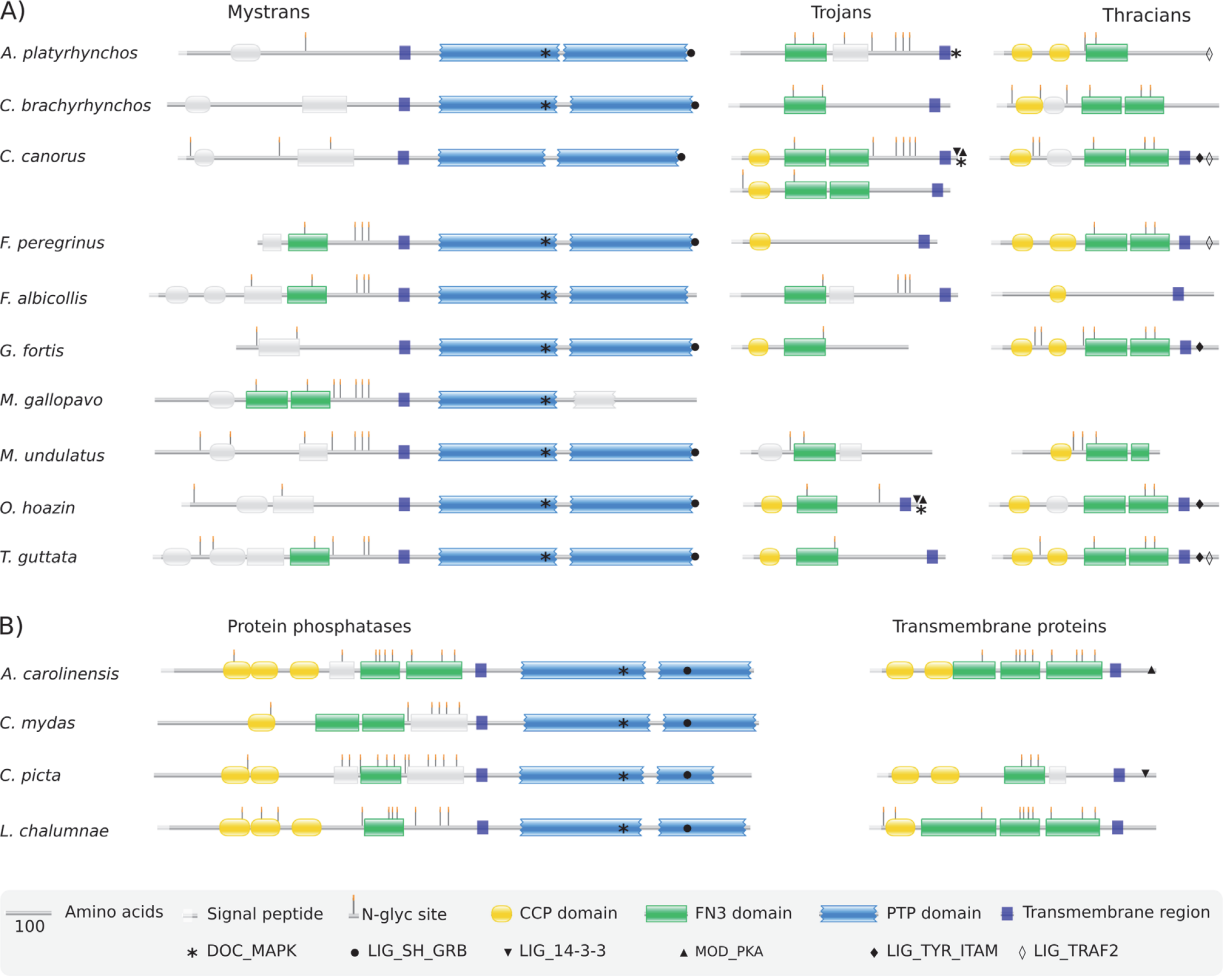
Trojan family proteins in other avian and non-avian species. Domain types, other topology properties and short functional motifs are shown in the legend. Domains presented in gray were predicted below threshold, but had the expected type, position and relative size. A) Avian species. B) Non-avian species.

To characterize the proteins encoded in the modeled genes, we predicted their topology organization. Avian Mystran, Trojan and Thracian homologues showed strong resemblance to their chicken counterparts (Fig. 3A). In some species, the proteins lacked some of the extracellular domains or had an extra CCP domain. Gene modeling was limited by some of the incomplete genomic sequences, which resulted in the prediction of several incomplete proteins. Also, we faced certain limitations when predicting domains in many of the species. For example, even though a region of gene conversion coding for a FN3 domain was identified between Trojan and Mystran in duck (Table 2), we were unable to detect the domain in Mystran. Therefore, for data completeness, we considered the predictions of the expected domains even if they appeared slightly below threshold.

**Table 2:** Gene conversion analyzes for the Trojan family in avian species.

Sequence I	Sequence II	BC KA P-value	Fragment in Sequence I	Fragment in Sequence II
MYS_ANAPL	TRO_ANAPL	3.93E-002	802–1176 (375)	607–1008 (402)
TRO_ANAPL	THR_ANAPL	9.20E-002	358–661 (304)	604–909 (306)
MYS_CORBR	TRO_CORBR	9.80E-002	919–3465 (2547)	706–1447 (742)
TRO1_CUCCA	TRO2_CUCCA	9.82E-002	376–1335 (960)	385–1290 (906)
TRO2_CUCCA	THR_CUCCA	1.17E-001	910–1435 (526)	1174–1495 (322)
MYS_GALGA	TRO_GALGA	5.86E-002	595–1641 (1047)	352–1383 (1032)
MYS_GEOFO	TRO_GEOFO	2.42E-001	61–531 (471)	274–771 (498)
MYS_OPHHO	TRO_OPHHO	1.98E-002	559–867 (309)	316–618 (303)

The gene converted fragments between sequence pairs (Sequence I and Sequence II) are given with respect to their unaligned offsets and lengths within each sequence. “BC KA P-values”: Bonferroni-corrected KA (BLAST-like P-values). Names combine Mystran (MYS), Trojan (TRO) or Thracian (THR) and the corresponding species abbreviation. Species: *A*. *platyrhynchos* (ANAPL), *C*. *brachyrhynchos* (CORBR), *C*. *canorus* (CUCCA), *G*. *gallus* (GALGA), *G*. *fortis* (GEOFO), *O*. *hoazin* (OPPHO).

All proteins with modeled cytoplasmic tails had a variety of intracellular SFM, but covering them all is beyond the scope of this paper. However, it is worth noting that the SFM found in chicken were also predicted in other species (Fig. 3A).

Mystrans showed the highest overall identity, due to their conserved cytoplasmic tails but had a high diversity within their extracellular regions (S2A, B and S3A Figs). The extracellular regions of Trojans were more homologous, especially at the CCP and the first FN3 domains. The homology decreased on the second FN3 domain and dropped even lower at the following, membrane-proximal part (S2C and S3B Figs). The most homologous proteins appeared to be the Thracians, which were highly similar at the second CCP and the FN3 domains (S2D and S3C Figs).

The two Trojan-family related genes in *A*. *carolinensis* were predicted to code for proteins with the same domain types as in chicken (Fig. 3B). The extracellular region of the lizard protein phosphatase was longer than that of Mystran in birds, having triplets of CCP and FN3 domains. The other gene coded for a transmembrane protein predicted to have a pair of CCP domains, three FN3 domains and a cytoplasmic tail similar in length to that of Thracian. The identified proteins were conserved mainly on the FN3 and the PTP domains (S3 Fig. D, E).

### Phylogenetic analyzes

**Fig 4:**
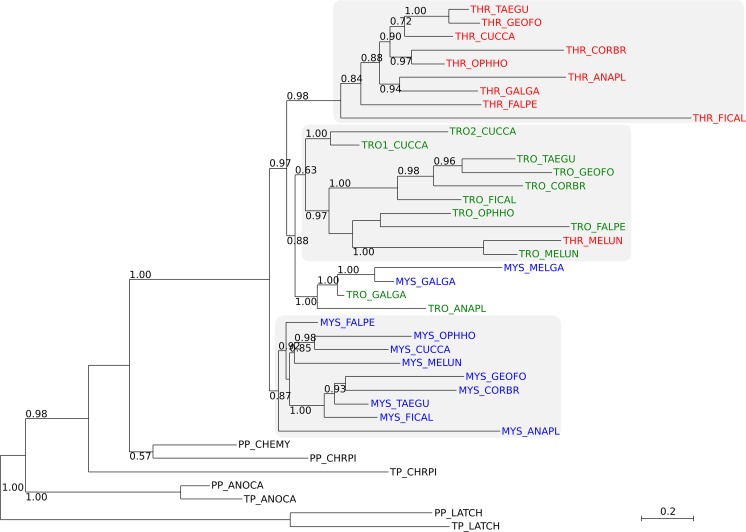
ML tree of the Trojan family members from all species. Mystrans are shown in blue, Trojans are shown in green and Thracians are shown in red. Major groups of homologues are enclosed within gray boxes. The tree is rooted to *L*. *chalumnae* and bootstrap values are indicated at nodes. Gene names combine the respective orthologue: Mystran (MYS), Trojan (TRO), Thracian (THR), Protein phosphatase (PP) or Transmembrane protein (TP) and species abbreviations. Avian species: *A*. *platyrhynchos* (ANAPL), *C*. *brachyrhynchos* (CORBR), *C*. *canorus* (CUCCA), *F*. *peregrinus* (FALPE), *F*. *albicollis* (FICAL), *G*. *fortis* (GEOFO), *M*. *gallopavo* (MELGA), *M*. *undulatus* (MELUN), *O*. *hoazin* (OPPHO), *T*. *guttata* (TAEGU); Non-avian species: *A*. *carolinensis* (ANOCA), *C*. *mydas* (CHEMY), *C*. *picta* (CHRPI), *L*. *chalumnae* (LATCH).

To investigate the evolutionary relationship between Trojan, Thracian and Mystran, we generated a maximum likelihood (ML) gene tree. The family members identified from avian species were analyzed along with the related members from reptiles and fish. After testing several substitution models in Phylogenetic estimation using ML (PhyML), we selected DCMut to construct the tree (see Materials and Methods for likelihood values and details). The tree was rooted to the proteins from *L*. *chalumnae* (Fig. 4).

Mystrans, Trojans and Thracians from avian species formed three major and one minor cluster (bootstrap values indicated in brackets). Almost all Mystrans were clustered together (87%), with the exception of those from *M*. *gallopavo* and *G*. *gallus*. A minor cluster was formed by Trojans and Mystrans (100%) from galloanseres species. Their atypical position in the tree is likely an effect of gene conversions between family members (see below). Mystran from *A*. *plathyrhynchos*, however, was clustered with the rest of the phosphatases. The rest of the Trojans formed another major group (63%) with Thracian from *M*. *undulatus* intertwined. It probably clustered there due to its incomplete genomic sequence, resulting in an incorrectly predicted peptide. The third major group was formed by the Thracian orthologues (98%).

The identification of a second Trojan gene in *C*. *canorus* is likely a result of gene duplication. Using the CODEML program from the Phylogenetic Analysis using Maximum Likelihood (PAML) suite, we estimated the duplication event to have occurred around 44.3 to 46.2 MYa. The estimates were calculated under local or global clock models of nucleotide substitution, calibrated with dates from the fossil record. The two Trojan genes formed their own minor subgroup (100%), within the clustered Trojans.

We then investigated all avian species for gene conversion events between their Trojan-like genes, using the GENECONV program. Focusing on silent sites only, we detected gene conversions in 6 species, listed in Table 2. From these, the largest converted fragment was found between *Mystran* and *Trojan* of *G*. *gallus*, which covers almost 350 extracellular amino acids. The second largest fragment was between the two Trojan genes from *C*. *canorus*, and accounted for about 300 amino acids. Gene conversions detected using default program settings are listed in S1 Table.

## Positive selection in birds

We investigated avian *Mystran*, *Trojan* and *Thracian* for evidence of positive evolutionary selection, often observed for immune-related genes. Each set of genes was analyzed by the CODEML program from the PAML suite. We compared model M8A (ω ratio varies between sites according to a beta distribution and ω_s_ = 1 is added to the beta distribution) versus M8 (adds a discrete class to the beta distribution ω_s_ > 1) to test the hypothesis of positive selection (Table 3). The ω site values estimated by the M8 model were considered for the further analyzes and graphical representation.

**Table 3:** Positively selected sites in the Trojan gene family.

Gene	LL test (M8A vs M8)	Sites with probability >90%
Mystran	2ΔL = 109.8P-value = 1.1E−25ω = 2.1 (16.5%)	4Q*, 6A*, 23H**, 24D, 28G*, 30Y*, 32G**, 33Y**, 34S, 44D**, 49R*, 54T**, 56A*, 84G*, 86D**, 89K, 90P*, 92Y, 163A, 165E, 166K**, 168A*, 169L**, 170D*, 172D*, 173G, 175I*, 179T, 181Q**, 188N, 194Q, 195T*, 251S*, 288S*, 290R*, 296A*, 300 K, 309R*, 322R*, 338Q, 344H*, 361T*, 366T*, 384S*, 397G*, 399P, 457S*, 462P*, 498G, 508A, 511S*, 531I*
Trojan	2ΔL = 13.5P-value = 0.00024ω = 1.5 (28.1%)	255A, 314T*, 316G**, 319H*, 321C, 324L, 326L, 327D*, 430S*
Thracian	2ΔL = 120.2P-value = 5.7E−28ω = 9.2 (7.2%)	26G*, 27A**, 28G*, 29A**, 30V*, 33K**, 34T**, 35E*, 36E**, 41E**, 48L, 87K**, 93G**, 94L*, 96A*, 190T**, 196A*, 465S*, 481A

Amino acids from chicken Mystran, Trojan and Thracian with Bayesian posterior probabilities to belong to site-class under positive selection are listed. Probability: >90%, >95% (*) or >99%(**), as inferred by Bayes-Empirical-Bayes (BEB).

For Mystran, M8 suggested 16.5% of sites to be positively selected with ω = 2.1, while for Trojan the sites accounted for 28.1%, with ω = 1.5. For Thracian, model M8 suggested 7.1% to be under positive selection, with ω = 9.2. Positively selected sites with probability over 90%, are listed in Table 3 and analyzed in details below.

**Fig 5:**
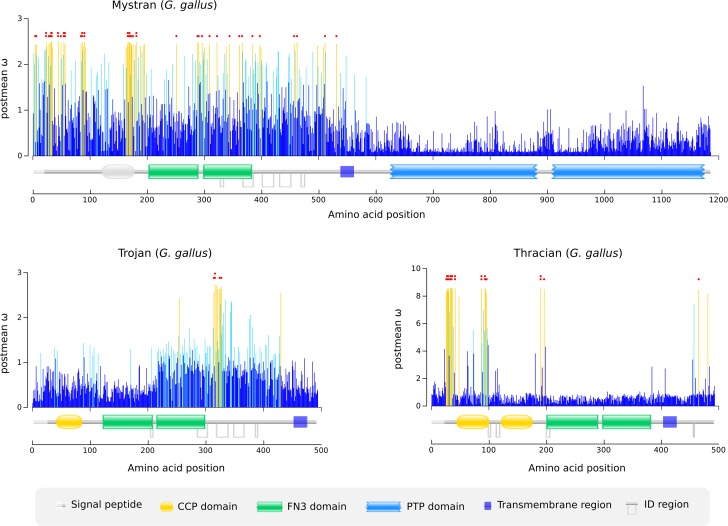
Evolutionary selection of the Trojan family members in chicken. Amino acid postmean ω values are mapped onto the protein topologies. Non selected sites are shown in blue, selected sites with probability below 90% are shown in light blue and selected sites with probability greater than 90% are shown in orange. Sites with probability greater than 95% and 99% are indicated by one or two red dots, respectively. Domain types and other topology properties are shown in the legend. The Mystran CCP domain is shown in gray scale, as it was predicted slightly below threshold, but had the expected position.

We plotted the post mean ω value of each amino acid of chicken Mystran, Trojan and Thracian against their positions in the polypeptide chain (Fig. 5). Mystran showed broad positive selection within its extracellular region, while Trojan had one major extracellular cluster of positively selected amino acids. Thracian had several extracellular patches of selected sites and two positively selected amino acids within the cytoplasmic tail.

Tests for evolutionary selection could be influenced by a heterogeneity within the MSA used, or by gene conversion between sequences. Therefore, we performed a set of side experiments, excluding divergent sequences or sequences bearing regions of gene conversions. Overall, these analyses showed similar results (S4 Fig. and S2 Table) to the ones presented in Fig. 5. See Materials and Methods section for details.

### Inter- and intramolecular co-evolution analyzes

**Fig 6:**
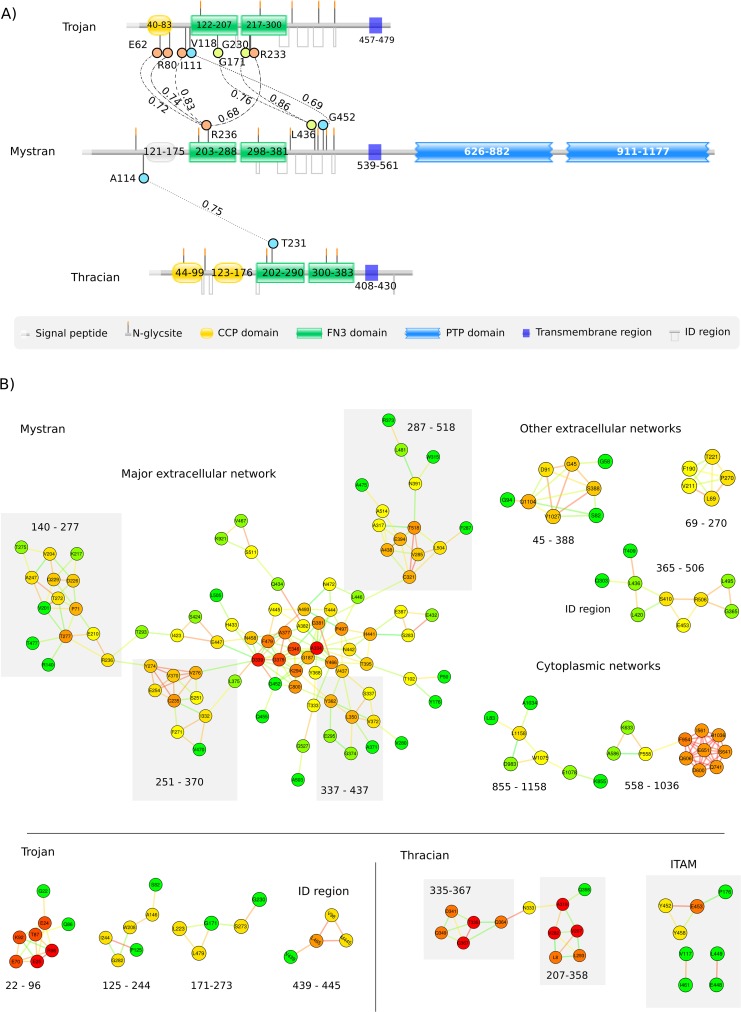
Co-evolutionary analyzes of Trojan family members. A) Intermolecular co-evolution between Trojan, Mystran and Thracian. Positions of co-evolving amino acids are mapped onto proteins topology from chicken. Correlation coefficients are indicated between each pair of residues. Coordinates on the polypeptide chain are indicated for each domain and transmembrane regions. Domain types and other topology properties are shown in the legend. B) Intramolecular co-evolution from chicken Mystran, Trojan and Thracian. Numerical values indicate the protein region to which the majority of network residues are confined.

To obtain evidence of functional interactions between the molecules, we analyzed the proteins for co-evolving amino acids (Fig. 6A). Three residues of Mystran were found as co-evolving with a total of 7 residues from Trojan. Mystran and Thracian had only one co-evolving pair of amino acids, while Trojan and Thracian had no significant co-evolving residues.

We then focused on intramolecular co-evolving amino acids, to search for functional co-dependence between residues within each protein (Fig. 6B). In Mystran, the largest co-evolutionary network incorporates over 90 nodes that formed numerous conjoined sub-networks. It contained almost exclusively extracellular amino acids, among which were many N-glycosylation sites and associated residues. The other major networks were not as broad and, with two exceptions, contained mainly extracellular residues. Amino acids from the membrane proximal extracellular region, rich in ID binding sites, were found to form their own network.

The number of co-evolving amino acids within Trojan was considerably lower, compared to that in Mystran. Nearly all of the co-evolving amino acids were extracellular, from which only two were associated with N-glycosylation sites. Networks were formed from residues belonging to the FN3 domains and from around the CCP domain. Similarly to Mystran, residues from the ID region were again found within networks, although considerably smaller.

In Thracian, we identified one major co-evolutionary network of extracellular residues, one of which is proximal to an N-glycosylation site. Most of the nodes belonged to the FN3 domains, mainly the second. Amino acids from the ITAM were found within several minor networks interconnected and/or linked to extracellular residues.

## Discussion

In this paper we present a novel gene family from chicken, that consists of the genes *Mystran*, *Trojan* and *Thracian*. We made extensive characterization of their deduced protein sequences, identified the family in other avian species and found related genes in non-avian species. We analyzed the phylogenetic relationship between the family members, estimated gene conversion events and dated one gene duplication. We have also determined patterns of evolutionary selection that have operated on the genes and identified co-evolving amino acid networks.

In chicken, the high expression of *Mystran*, *Trojan* and *Thracian* genes in macrophages is in consent with the previously reported tissue distribution of Trojan [16]. Trojan is a leukocyte-specific molecule and we can expect the other family members to be related to immune system function, as well. Detailed tissue expression analyzes of the family will be among the primary objectives of our further studies.

Chicken *Mystran*, *Trojan* and *Thracian* code for surface proteins with CCP and FN3 domains within their extracellular regions. Such domain types are known to mediate molecular associations in *cis*, *trans* or in a combined fashion, as has been shown for the IL-2 receptor complex [17]. Hence, the extracellular topology of the Trojan protein family suggests an ability for interaction with other cell surface partners or ligands. These domains were also found in the proteins from the other avian and non-avian species, implying their functional importance. The proposed ability for protein-interaction is further supported by the presence of extracellular ID binding sites. Like in the case of the immune-related CD44, many ID regions are extracellular [18]; they are also believed to mediate protein interactions and often represent flexible areas between domains [19]. Indeed, we identified the ID binding sites mainly within regions where no known domains were predicted to exist. Therefore, the ID regions may complement the molecular function of the CCP and FN3 domains or have a role on their own in the process of protein binding.

The Trojan family members have similar extracellular regions, but dissimilar intracellular parts, that bear signatures of signaling potential. The pair of PTP domains in Mystran outlines the molecule as the only family member capable of direct catalytic activity. The overlapping cytoplasmic SFM of Trojan indicate an indirect signaling potential of the molecule via the association of intracellular partners. Among the cytoplasmic SFM of Thracian was an ITAM, a commonly found motif in transmembrane proteins of the immune system [20]. The short ID binding region next to it, may mediate the functional interactions of the motif with other molecules [21]. Considering the presence of Thracian transcript in macrophages, the ITAM makes us further suspect a role of it related to immunity. Overall, these data indicate that the Trojan family members have potential for protein interactions and downstream signaling. The SFM hint towards possible intracellular partners of Mystran, Trojan and Thracian that represent an intriguing aspect of our further studies. The nature of such interactions, the triggered cytoplasmic cascades and their cellular role are yet to be elucidated experimentally. The prediction of the same SFM in avian species other than chicken highlights the potential functional importance of the molecules.

In our ML analyzes, the family members from reptiles and fish naturally formed an outgroup, helping us root the tree. Although we found homologues in the coalecanth, we were unable to find related genes in other fish, like *D*. *rerio*. Considering that the coalecanth is evolutionarily close to reptiles [22], we could expect the genes to have come into existence after the emergence of ray-finned fish. Our searches however, did not identify homologous sequences in *X*. *tropicalis*, which could be due to an incompletion of the sequences databases at the time the searches were done. No evidence of the family in mammals was found, suggesting a gene loss. The reasons behind such an event, as well as the identification of possible functionally-related genes in mammals are intriguing targets for our future analyzes.

Most of the avian orthologues grouped into three separate clusters, as was expected. The two major clusters of Trojans and Thracian are likely a result of a recent duplication in birds. However, the orthologs do not exactly recapitulate the species tree [23] and some appear similar to another family member from the same species. This is likely a result of gene conversions and as our data showed, such events have indeed occurred in several species. The most notable conversion was the one between Mystran and Trojan in chicken, which accounts for the remarkably high similarity between their extracellular regions. As a result, this placed the two genes next to each other in the tree, instead of their respective clusters. Therefore, the phylogenetic tree is a mix of gene duplication and gene conversion which results in its overall ladder-like shape.

If the gene conversion between *Mystran* and *Trojan* has provided a functional advantage, the benefit would have stemmed from the partial identity between the two proteins. This raises the intriguing possibility that the extracellular regions of Trojan and Mystran may be capable of associating with the same partner. Such interaction may result in a partner-binding competition and may represent a means of functional regulation of the molecules. The existence of such a process is an exciting target for future investigation, as it may underpin a co-dependence of Mystran and Trojan.

Cuckoo was the only species with 4 family members, with the second Trojan gene likely being the result of a duplication event that occurred 44–46 MYa. The calculated age is relatively distant, suggesting that the identified fourth family member is an actual gene and not a sequencing anomaly. A conversion was found between the two Trojan genes, making the proteins very similar in their extracellular regions. The functional advantage of an extra Trojan is also a target of future studies, as we will look for a similar gene organization in other avian species.

Evolutionary pressure to adapt has probably played a major role in the diversification of Mystran, Trojan and Thracian genes, as seen in the tree. Indeed, we found evidence of positive evolutionary selection for all the family members. For Mystran, the extensive positive selection within its extracellular region has probably been a response to changes in its ligand or interacting molecules. The positively selected residues found outside the domains likely provided an overall protein adaptation and indirect flexibility for the structured regions. However, the “spikes” of positive selection within the domains imply, that certain structural or recognition adjustments were required. In contrast, the extremely conserved cytoplasmic tail of Mystran, hints that the downstream signaling mechanism had to remain unchanged. Therefore, Mystran is a protein for which intense extracellular adaptation of molecular interaction is coupled to intracellular preservation of function. This overall evolutionary pattern appears very similar to the selection described for another rPTP, the common leukocyte antigen CD45 [9,24]. Fast evolving molecules tend to interact with other rapidly evolving molecules [25], raising the question of what the partner of Mystran could be. As already proposed, we can expect Mystran to interact with a ligand or some other cell surface protein. If the phosphatase is immune-related, the extensive positive evolutionary selection may have been in response to pathogen challenges.

For Trojan, the relatively low number of positively selected amino acids was probably due to functional constraints or less adaptation challenges. The single cluster of selected residues falls within a region rich in ID binding sites, further pointing towards the importance of that area. The selection has probably lead to an adaptation of the binding properties of the region and the protein as a whole. ID regions have indeed been shown to be highly mutable and evolving, probably due to the lack of structural restraints [26]. Therefore, the relative conservation of the domains may have been compensated by the intensive adaptation of this region. The lack of positive selection within the cytoplasmic tail, implies that its putative signaling mechanism did not require adjustments.

For Thracian, the three extracellular patches of positively selected sites probably provided adaptive flexibility for the adjacent domains. The conservation of the domains was likely due to functional constraints or simply no requirements for fine-tuning. The positively selected sites within the cytoplasmic tail indicate some adaptation of its intracellular signaling potential.

The possibility of a functional interaction between Mystran and Trojan was suggested by the identified intermolecular co-evolving amino acids. The pair of co-evolving amino acids between Mystran and Thracian hints towards a co-dependent function of them, as well. This could place the phosphatase in the middle of the intermolecular co-evolutionary events, where the family members may even function in concordance via Mystran. The actual existence of of such co-dependence and whether it involves a physical interaction between the proteins has yet to be determined experimentally. Amino acids from separate domains, co-evolving with the same residue of another protein, may be a sign of domain co-dependence [27]. Therefore, we could expect a functional correlation between the domains of Trojan, as several of their residues are co-evolving with the same amino acid from Mystran.

In Mystran, the numerous connected constellations of co-evolving residues probably provided a coordinated overall adaptation of the extracellular region. The N-glycosylation sites among them indicate a global adjustment of the sugar frame, probably of structural advantage. The smaller networks are likely responsible for the localized adaptation of distinct regions of the protein. The two major cytoplasmic networks of Mystran were relatively small, likely due to conservation of the signaling mechanism.

Identifying co-evolving amino acids from a region of no assessed domains hints towards some functional significance of the region [27]. Finding such residues from the ID binding sites of Mystran and Trojan, further supports the proposed functional importance if these regions.

The largest network in Trojan was formed mainly by residues around the CCP domain, likely due to functional constraints. Such a co-evolutionary pattern probably helps to maintain the conformational and functional stability of a domain [28]. Residues from the two FN3 domains were found within the other networks, further hinting towards their co-dependent function. No networks were found for the cytoplasmic tail, probably due to conservation of its hypothesized signaling mechanism.

In Thracian the largest evolutionary network consisted of residues confined to the pair of FN3 domains, suggesting an active process of co-dependent adaptation. In the cytoplasm, finding amino acids of the ITAM as mutually co-evolving underlines the significance of this SFM.

The identified extracellular co-evolutionary networks within Mystran, Trojan and Thracian hint towards a yet another functional possibility. As has been described for TLRs [10], intramolecular co-evolving amino acids can be linked to the ability of a molecule to form homodimers. Therefore, in addition to the suggested interaction between the family members, Mystran, Trojan and Thracian may also homodimerize. The existence and functional means of such interaction is an intriguing topic of further investigation.

## Conclusions

The previously described Trojan protein has been predicted to be part of a novel chicken gene/protein family. Here, we characterize the other Trojan-like family members from chicken and show that the family exists in other birds, as well as reptiles and fish. The phylogenetic analysis revealed a step-wise segregation between the homologues across avian species, a result of gene conversion events. We demonstrate that positive evolutionary selection has acted predominantly on the extracellular regions of the family members. In contrast, almost no positively selected sites were found within their intracellular regions. Therefore, the opposing evolutionary selections combined an environment-driven extracellular adaptation with preservation of the cytoplasmic signaling mechanism. The predicted topology of the proteins hints towards extracellular ligand/partner interactions, that are yet to be identified. Our co-evolutionary analyzes suggested a functional co-dependence between the family members, that may involve their physical association. However, further studies are required to determine the exact functional role of the family and their interacting partners.

## Materials and Methods

### Identification of the Trojan gene family in chicken

The Trojan family was identified from the chicken genome database annotations (v4.0) at the NCBI on chromosome Z: *Mystran* (8862119..8883723, GeneID: LOC100858919), *Trojan* (8883937..8889333, GeneID: 427414) and *Thracian* (8891672..8898353, GeneID: LOC100858953). The bordering genes are *RUSC2* (GeneID: 431657) and *TESK1* (GeneID: 429878). The CDS and amino acid sequences of Mystran (RefSeq: XM_003642970.2) and Thracian (RefSeq: XM_003642971.2) were downloaded from the database for the further analyzes. For Trojan, the database annotated two transcriptional variants: *Trojan-X1* (RefSeq: XM_003642914.2) and *Trojan-X2* (RefSeq: XM_004937133.1). Using the online bl2seq tool http://blast.ncbi.nlm.nih.gov/) to compare the two sequences, *Trojan-X1* was found to be 99% identical to the Trojan clone reported previously [16]. We used the cDNA and deduced amino acid sequences of the identified Trojan clone (GenBank: FN643572.1) for all subsequent analyzes.

### *In silico* expression analyzes

The Ensembl (http://www.ensembl.org/) genome browser (release 75 – February 2014) was searched with the NCBI gene coordinates of Mystran (chicken Z: 8862119..8883723), Trojan (chicken Z: 8883937..8889333) and Thracian (chicken Z: 8891672..8898353). The following RNASeq alignments were selected: brain, breast, cerebellum, fibroblasts, embryo, heart, kidney, liver, macrophages, testes, somites. Their read values were plotted using Gnumeric spreadsheet (https://projects.gnome.org/gnumeric/).

### Homology sequence searches

The Mystran, Trojan and Thracian amino acid sequences from chicken were used in BLASTP and BLAT searches against the genomic/translated databases at NCBI, UCSC (University of California Santa Cruz: http://genome.ucsc.edu/) and EBI (European Bioinformatics Institute: http://www.ebi.ac.uk/). The genomic sequences to which we found similarity hits were collected as chromosomal regions or whole scaffolds, depending on the level of database completion.

### Gene modeling and prediction in avian and non-avian species

The CDS of chicken Mystran, Trojan and Thracian were aligned to the collected avian genomic sequences using Spidey (http://www.ncbi.nlm.nih.gov/spidey/), driven by UniPro Ugene [29]. This provided an estimate of the genomic regions to be used in the gene modeling step. If a gene spanned more than one scaffold, scaffolds were joined, following the gene orientations in chicken as a reference. Gene homologues were modeled after the corresponding proteins from chicken, using GeneWise [30] (http://www.ebi.ac.uk/Tools/psa/genewise/), with the following settings: global mode, modeled splice sites, synchronous model, algorithm 623.

Scaffold GL343585.1 from *A*. *carolinensis* (Table 1) that showed homology to the chicken Mystran, Thracian and Trojan, was processed by GENSCAN [31] web tool (http://genes.mit.edu/GENSCAN.html). This predicted two lizard genes homologous to the Trojan family from the regions. Their deduced amino acid sequences were run in a second similarity search that gave homology hits to genomic sequences of other non-avian species. We then modeled these other non-avian genes after the proteins from *A*. *carolinensis*, using the same strategy as for the avian species. GeneWise settings were the same as above, except for *C*. *mydas* where a flat model was used.

The exon/intron organization of the modeled genes was determined by aligning their CDS to the genomic sequences. Graphical representation of the genes and scaffold assemblies was rendered by the GenomeTools software package [32].

### Mammalian genome searches

We created HMM3 (hidden Markov models) profiles from the sequences of Mystrans, Trojans and Thracians by HMMER3, included in UniPro Ugene. We performed searches in several mammalian genomes between the genes *RUSC2* and *TESK1*. Among the species searched were *Homo sapiens* (RefSeq: NC_000009.11) and *Mus musculus* (RefSeq: NC_000070.6).

### Gene conversion analyzes

MSA of the family members CDS were generated for each species by Clustal Omega [33], driven by SeaView [34]. The alignments were then analyzed by GENECONV [35], with mismatch a penalty of 1 (option: /g1).

For a pair of CDS, amino-acid polymorphisms may have arisen as a result of strong evolutionary selection, becoming clustered in the alignment. This would artificially elevate the significance of fragments detected elsewhere by GENECONV. For this, the program provides the option to focus on silent polymorphic sites only and examine the synonymous changes within codons. Given the positive evolutionary selection identified by our other experiments we decided to use this approach, too. The avian family members CDS were codon aligned following their translated MSA, by PAL2NAL [36](http://www.bork.embl.de/pal2nal/). The alignments were then analyzed for recombination by GENECONV, considering only silent-site polymorphisms (option: /r).

### Estimation of the time of gene duplication

A tree based on the most recent avian phylogeny [23] was constructed for the Trojan orthologs from the avian species. Fossil calibration values were inferred from the species divergence times, obtained from the TIMETREE web-site (http://www.timetree.org/). The duplication time of the two Trojan genes from *C*. *canorus* was then estimated by CODEML, part of the PAML suite [37]. Global or local clock models (clock = 5 and clock = 6, respectively) were used, each in two runs of CODEML. In the first run, the κ (the transition/transversion ratio) and ω (the non-synonymous/synonymous ratio) were estimated. In the second run, the duplication time was estimated with fixed values for κ and ω.

### Amino acid sequence analyzes

Domain organization of the proteins was predicted using the SMART (simple modular architecture research tool: http://smart.embl-heidelberg.de/) database in *normal* mode with detection for *outlier homologues*, *PFAM domains* and *signal peptides*. Putative serine, tyrosine and threonine phosphorylation sites were predicted with NetPhos (http://www.cbs.dtu.dk/services/NetPhos/). N-glycosylation sites were predicted by NetNGlyc (http://www.cbs.dtu.dk/services/NetNGlyc/). Searches for short functional motifs were performed at ELM (Eucaryotin Linear Motif: http://elm.eu.org/) [38]. Schematic representation of the overall protein topology was done by the domain images generator tool at PFAM (http://pfam.sanger.ac.uk/generate_graphic/), while cytoplasmic tails were drawn by PROTTER (http://wlab.ethz.ch/protter/).

Pairwise alignments between the chicken family members were performed by Jalview [39] with default settings. MSA were performed independently for each family member from avian or non-avian species by Clustal Omega, driven by SeaView. We used Aline [40] to visualize the MSA in a similarity color code and to annotate the domain organization of the reference sequence for each alignment. Distance matrix was generated for each set of MSA in UniPro Ugene, using identity distance algorithm and percent profile mode.

Disordered regions in chicken Mystran, Thracian and Trojan were predicted at the Dismeta server (http://www-nmr.cabm.rutgers.edu/bioinformatics/disorder/), which utilizes a vast variety of disorder prediction tools. Potential protein binding regions were identified by ANCHOR [41] (http://anchor.enzim.hu/).

### Phylogenetic analyzes

All identified family members from avian and non-avian species were analyzed jointly for their phylogenetic relationship at amino acid level. Sequences were aligned by Clustal Omega and subjected to maximum likelihood analyzes by PhyML [42]. Both programs were driven by SeaView, which was also used to graphically depict the obtained tree. The following substitution models were tested: LG, WAG, Dayhoff, JTT, Blosum62, DCMut and VT. DCMut had best likelihood value (lnL = -48330.3) and therefore we further optimized the program settings to use subtree pruning and regrafting (SPR) in the tree searching operations. The ML tree was derived from this optimized run of the program, in with DCMut yielded a lnL = -48333.4.

### Tests for positive selection

We created separate codon alignments of avian Mystran, Thracian and Trojan CDS by PAL2NAL. Alignments were based on the protein MSA generated earlier and were independent for each gene set. We then analyzed the codon alignments in conjunction with trees based on the latest avian phylogeny [23] by the CODEML program, part of the PAML suite. The three sets of avian genes were tested independently of each other by a pair of models of evolutionary selection: M8A (beta & ω_s_ = 1: fix omega = 1, omega = 1, NS sites = 8) versus M8 (beta & ω: p0, p1, p, q, ω_s_ > 1, NS sites = 8). The tests were compared (2ΔL) in Gnumeric and the *chidist* formula (survival function of the chi-squared distribution) was used to calculate their likelihood estimates (P-value). The postmean ω values estimated by Bayes Empirical Bayes (BEB) of the selected model were plotted in Gnumreic using the amino acid numbering of chicken Mystran, Thracian and Trojan.

Since we observed a certain level of heterogeneity of the MSA used above, we did alternative runs of CODEML, with the most divergent sequences omitted. These were *MYS_ANAPL*, *TRO_FALPE* and *THR_FICAL* from the Mystran, Trojan and Thracian gene sets, respectively. We also performed two extra analyzes of Trojan, excluding *TRO2_CUCCA—*a sequence bearing a gene conversion fragment with *TRO1_CUCCA*. Analysis was done with or without *TRO_FALPE* present in the MSA.

### Co-evolution analyzes

We used CAPS2 (Coevolution Analysis using Protein Sequences 2: http://caps.tcd.ie/) [43] with default settings to identify co-evolutionary patterns. Intra- and intermolecular co-evolving amino acids were detected using the MSA generated at the amino acids sequence analyzes step. All reported amino acid sites refer to the positions in Mystran, Trojan and Thracian from chicken. Intramolecular co-evolutionary networks were visualized by Cytoscape [44], while intermolecular co-evolving amino acids were mapped manually onto protein topology.

## Supporting Information

S1 Fig Trojan family genes in avian species and non-avian species. Regions used for gene prediction are indicated as blank boxes showing the gene direction. Exon organization of the predicted genes is presented as filled fragments. Scaffolds are shown as light-gray pointed boxes, indicating their assembly and orientation. Gray triangles on scaffolds’ sides indicate preceding, successive and connecting sequence segments. Gene names combine the respective homologue: Mystran (MYS), Trojan (TRO), Thracian (THR), Protein phosphatase (PP) or Transmembrane protein (TP) and the corresponding species abbreviation. A) Avian species: *A*. *platyrhynchos* (ANAPL), *C*. *brachyrhynchos* (CORBR), *C*. *canorus* (CUCCA), *F*. *peregrinus* (FALPE), *F*. *albicollis* (FICAL), *G*. *fortis* (GEOFO), *M*. *gallopavo* (MELGA), *M*. *undulatus* (MELUN), *O*. *hoazin* (OPPHO), *T*. *guttata* (TAEGU). B) Non avian species: *A*. *carolinensis* (ANOCA), *C*. *mydas* (CHEMY), *C*. *picta* (CHRPI), *L*. *chalumnae* (LATCH).
(PDF)

S2 Fig Distance matrix of avian Mystran, Trojan and Thracian MSA. Amino acid sequence identity is shown as a percentage. Names combine Mystran (MYS), Trojan (TRO) or Thracian (THR) and the corresponding species abbreviation. Species: *A*. *platyrhynchos* (ANAPL), *C*. *brachyrhynchos* (CORBR), *C*. *canorus* (CUCCA), *F*. *peregrinus* (FALPE), *F*. *albicollis* (FICAL), *G*. *fortis* (GEOFO), *M*. *gallopavo* (MELGA), *M*. *undulatus* (MELUN), *O*. *hoazin* (OPPHO), *T*. *guttata* (TAEGU). A) Mystrans; B) Mystrans, excluding cytoplasmic tails; C) Trojans; D) Thracians;
(PDF)

S3 Fig Orthologues MSA from avian and non-avian species. Amino acid similarity is indicated by a color saturation scale. The domain organization of the first sequence is shown on top as a reference; SP (gray): signal peptide, CCP (orange): complement control protein domain, FN3 (green): Fibronectin type III domain, PTP (light blue): protein tyrosine phosphatase domain, TM (blue): transmembrane region. Names combine Mystran (MYS), Trojan (TRO), Thracian (THR), Protein phosphatase (PP) or Transmembrane protein (TP) and the corresponding species abbreviation. Avian species: *A*. *platyrhynchos* (ANAPL), *C*. *brachyrhynchos* (CORBR), *C*. *canorus* (CUCCA), *F*. *peregrinus* (FALPE), *F*. *albicollis* (FICAL), *G*. *fortis* (GEOFO), *M*. *gallopavo* (MELGA), *M*. *undulatus* (MELUN), *O*. *hoazin* (OPPHO), *T*. *guttata* (TAEGU). Non-avian species: *A*. *carolinensis* (ANOCA), *C*. *mydas* (CHEMY), *C*. *picta* (CHRPI), *L*. *chalumnae* (LATCH). A) MSA of avian Mystrans; B) MSA of avian Trojans; C) MSA of avian Thracians; D) MSA of non-avian Protein phosphatases; E) MSA of non-avian Transmembrane proteins.
(PDF)

S4 Fig Alternative analyzes of the evolutionary selection of the Trojan family members in chicken. Amino acid postmean ω values are mapped onto the protein topologies. Non selected sites are shown in blue, selected sites with probability below 90% are shown in light blue and selected sites with probability greater than 90% are shown in orange. Sites with probability greater than 95% and 99% are indicated by one or two red dots, respectively. Domain types and other topology properties are shown in the legend. The Mystran CCP domain is shown in gray scale, as it was predicted slightly below threshold, but had the expected position. A) *Mystran*, *Trojan* and *Thracian* analyzed without the sequences that appeared too divergent (*MYS_ANAPL*, *TRO_FALPE* and *THR_FICAL*, respectively). B) *Trojan* analyzed with *TRO2_CUCCA* omitted. C) Trojan analyzed with *TRO2_CUCCA* and *TRO_FALPE* omitted.
(PDF)

S1 Table Gene conversion analyzes for the Trojan family in avian species, using default settings. The gene converted fragments between sequence pairs (Sequence I and Sequence II) are given with respect to their unaligned offsets and lengths within each sequence. “BC KA P-values”: Bonferroni-corrected KA (BLAST-like P-values). Names combine Mystran (MYS), Trojan (TRO) or Thracian (THR) and the corresponding species abbreviation. Species: *A*. *platyrhynchos* (ANAPL), *C*. *brachyrhynchos* (CORBR), *C*. *canorus* (CUCCA), *F*. *peregrinus* (FALPE), *F*. *albicollis* (FICAL), *G*. *fortis* (GEOFO), *M*. *gallopavo* (MELGA), *M*. *undulatus* (MELUN), *O*. *hoazin* (OPPHO), *T*. *guttata* (TAEGU).
(PDF)

S2 Table Positively selected sites in the Trojan gene family, identified by alternative analyzes. Amino acids from chicken Mystran, Trojan and Thracian with Bayesian posterior probabilities to belong to site-class under positive selection are listed. Probability: >90%, >95% (*) or >99%(**), as inferred by Bayes-Empirical-Bayes (BEB).
(PDF)
