# Supplementary figures and images for: Characterization of the Avian Trojan Gene Family Reveals Contrasting Evolutionary Constraints
Source: PLoS One. 2015 Mar 24;10(3):e0121672. doi: 10.1371/journal.pone.0121672 (PMC4372362; doi:10.1371/journal.pone.0121672)

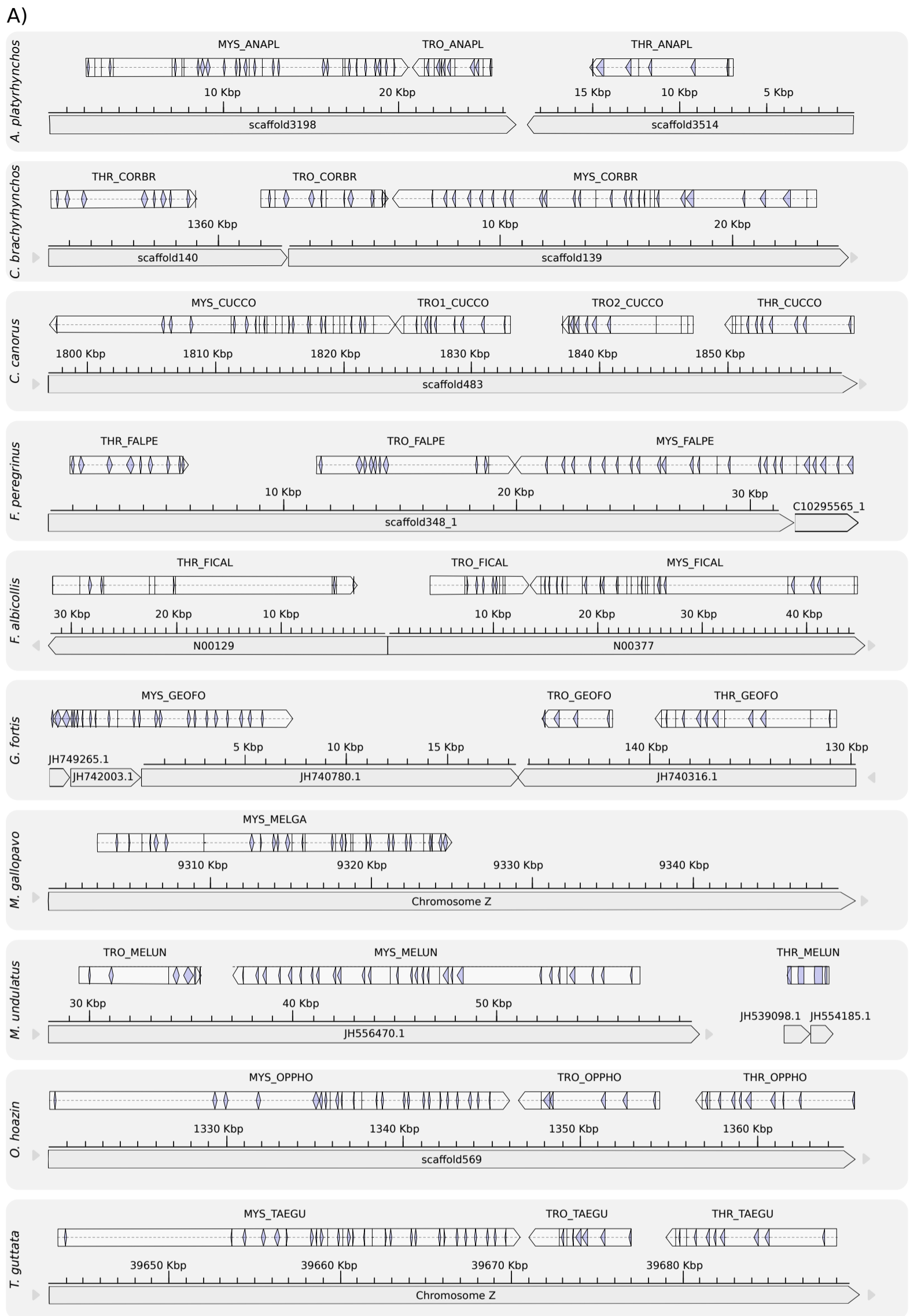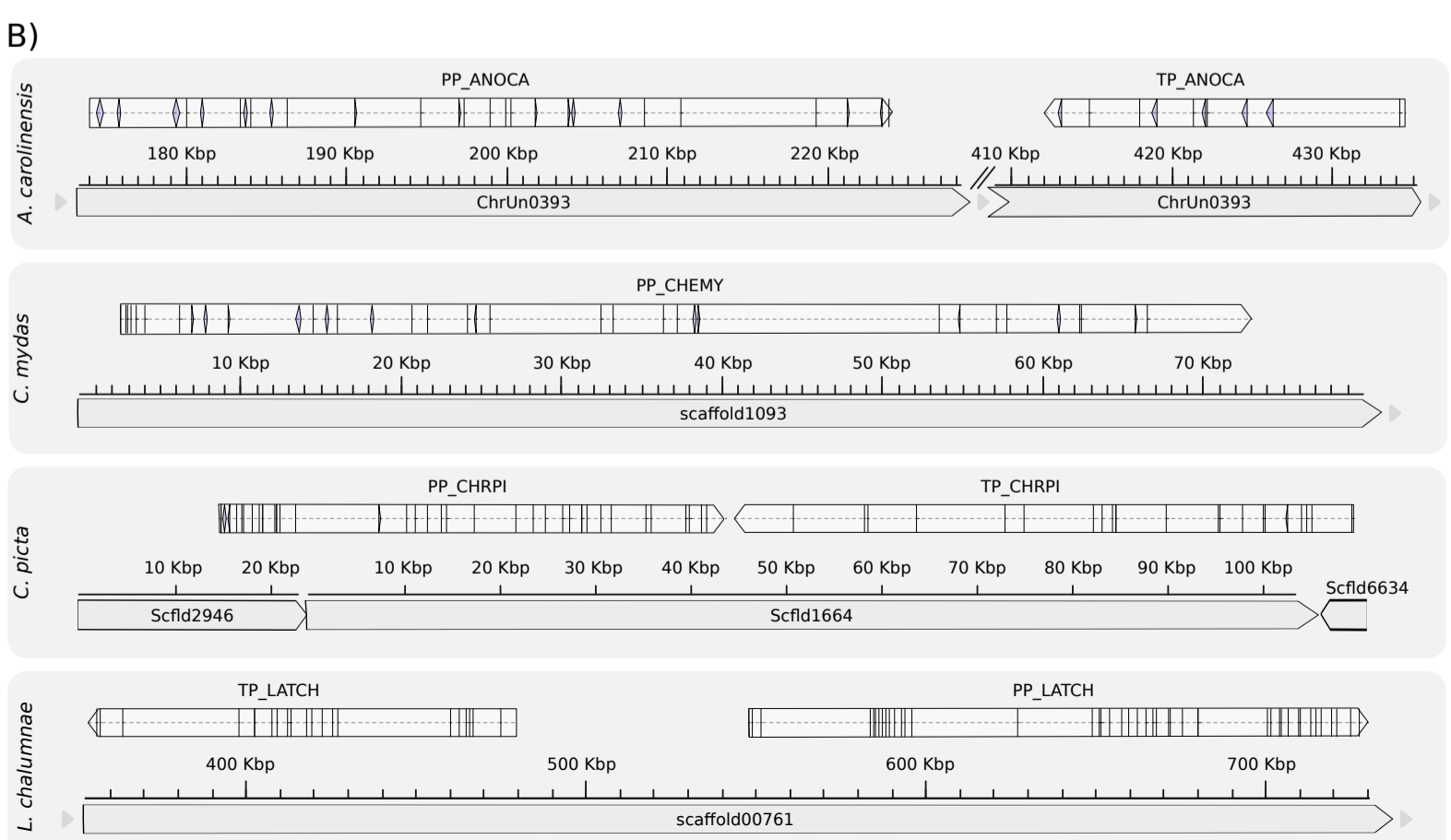

Supplement: S1 Fig — Regions used for gene prediction are indicated as blank boxes showing the gene direction. Exon organization of the predicted genes is presented as filled fragments. Scaffolds are shown as light-gray pointed boxes, indicating their assembly and orientation. Gray triangles on scaffolds’ sides indicate preceding, successive and connecting sequence segments. Gene names combine the respective homologue: Mystran (MYS), Trojan (TRO), Thracian (THR), Protein phosphatase (PP) or Transmembrane protein (TP) and the corresponding species abbreviation. A) Avian species: A. platyrhynchos (ANAPL), C. brachyrhynchos (CORBR), C. canorus (CUCCA), F. peregrinus (FALPE), F. albicollis (FICAL), G. fortis (GEOFO), M. gallopavo (MELGA), M. undulatus (MELUN), O. hoazin (OPPHO), T. guttata (TAEGU). B) Non avian species: A. carolinensis (ANOCA), C. mydas (CHEMY), C. picta (CHRPI), L. chalumnae (LATCH). (PDF) [file pone.0121672.s001.pdf]

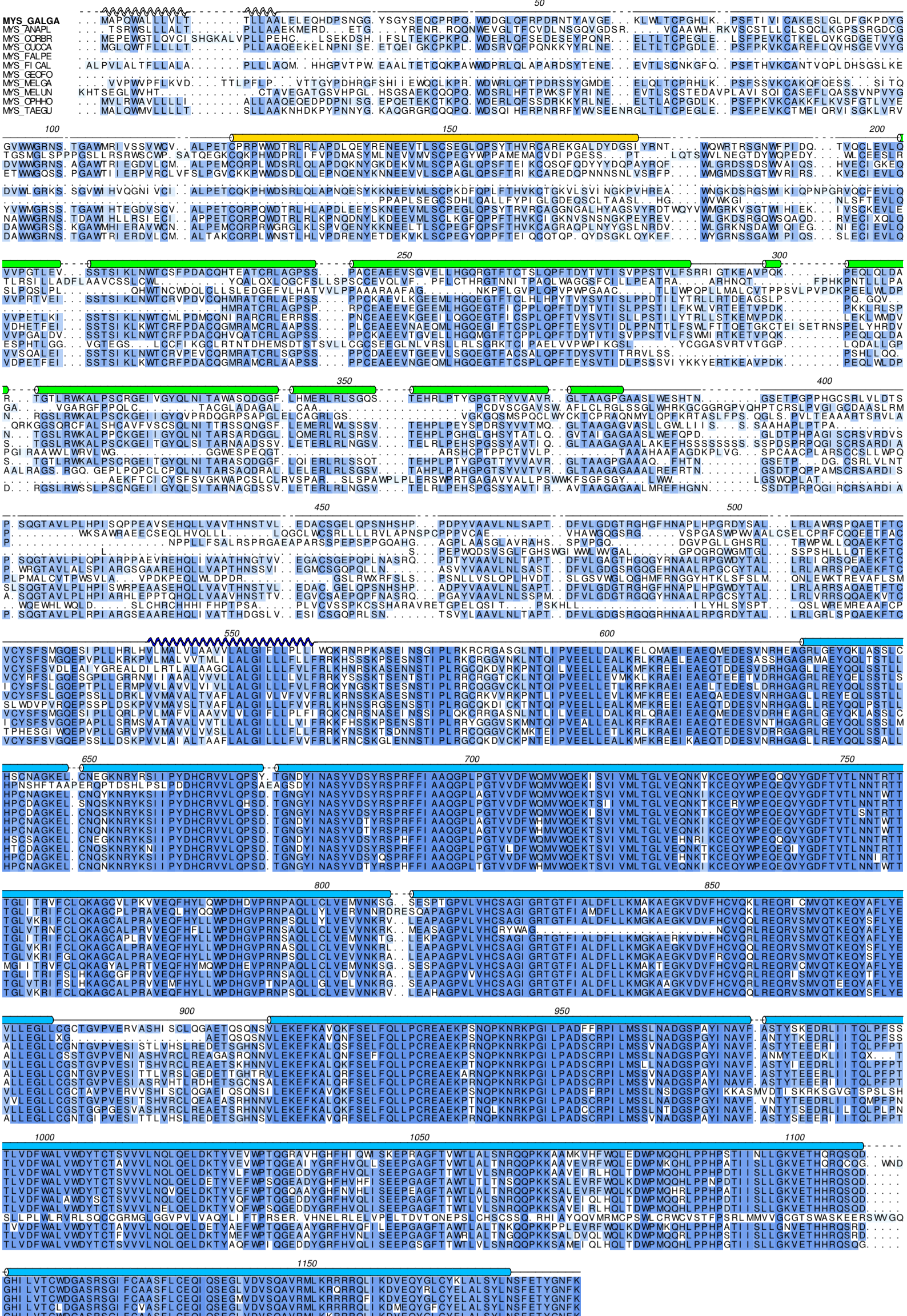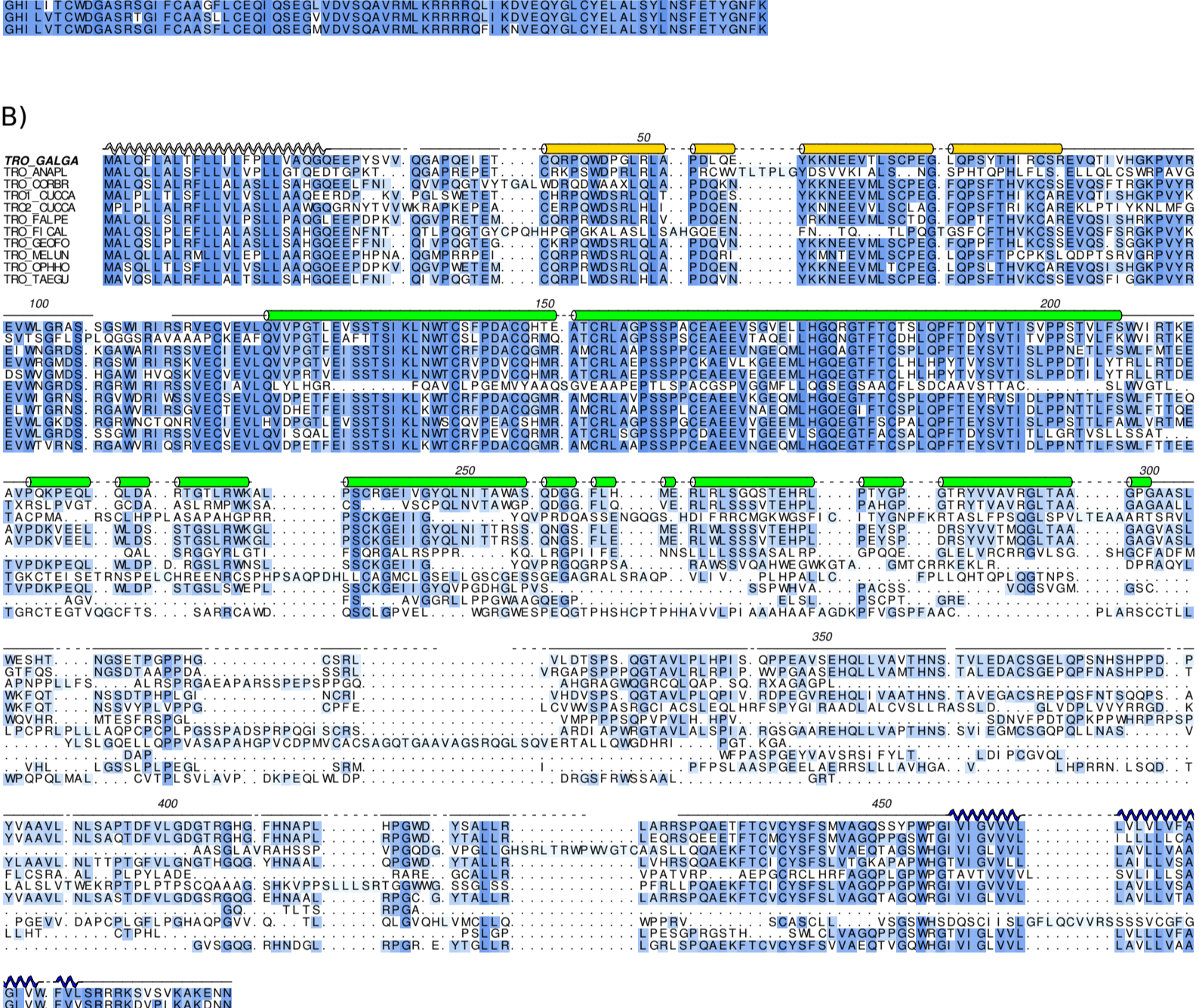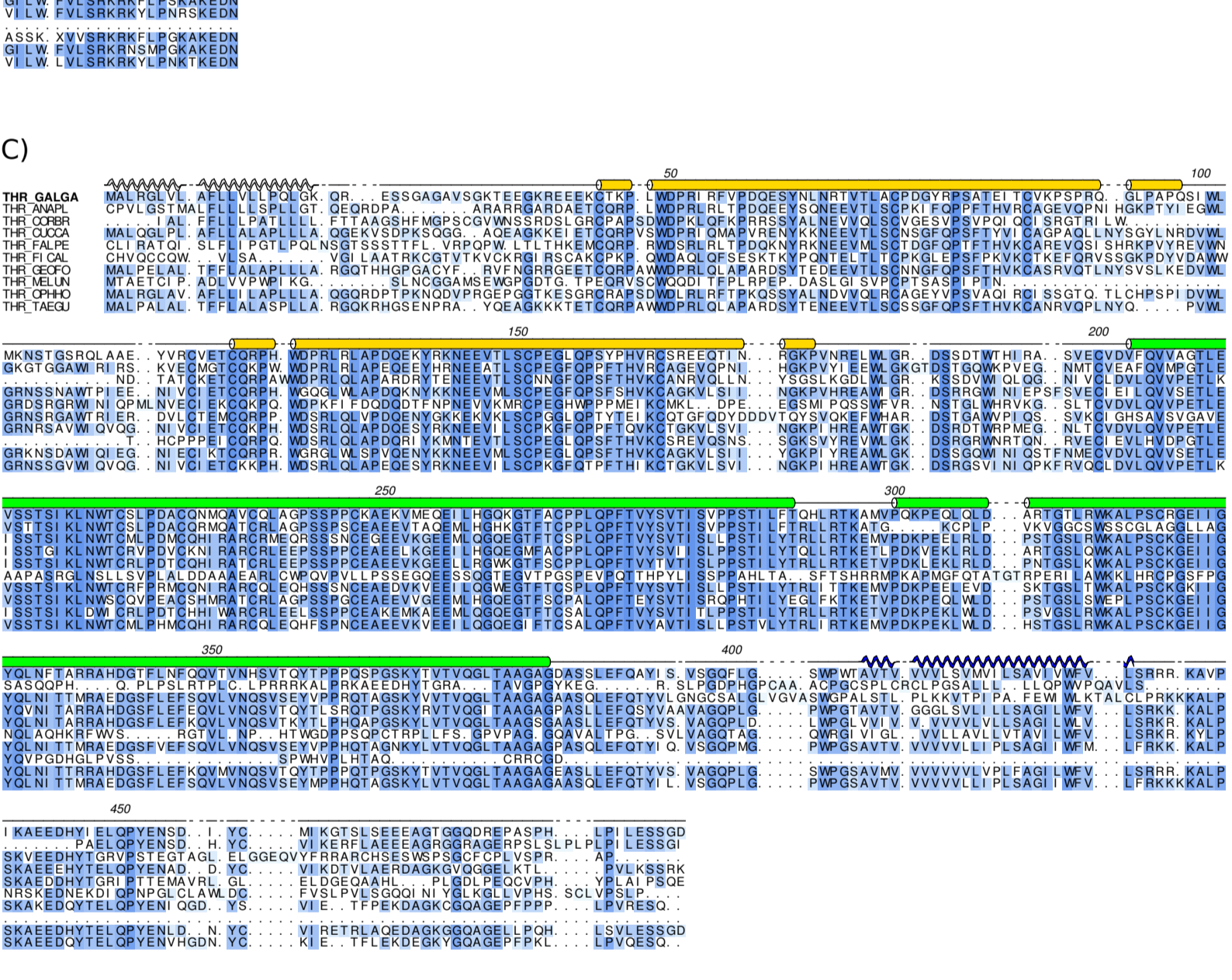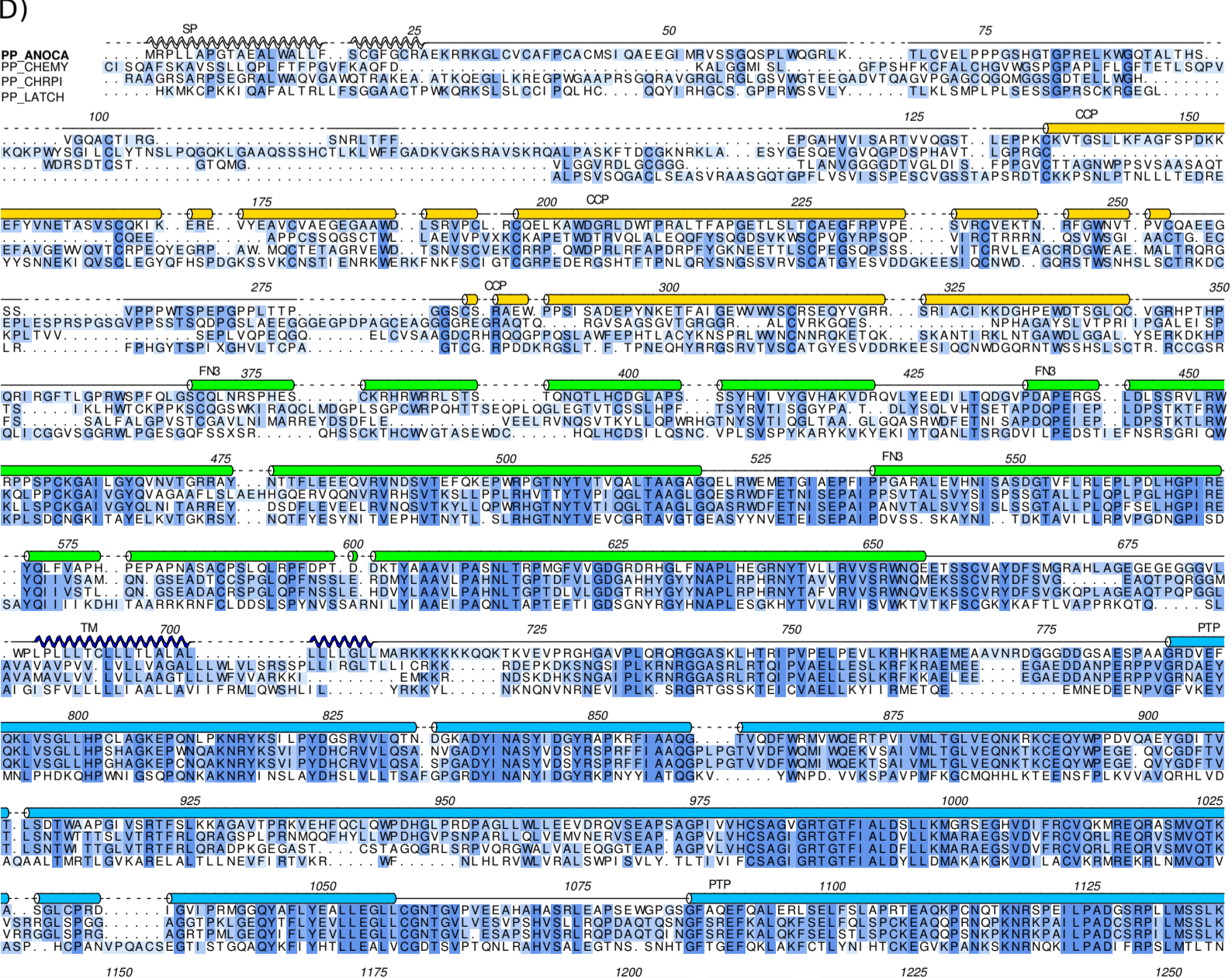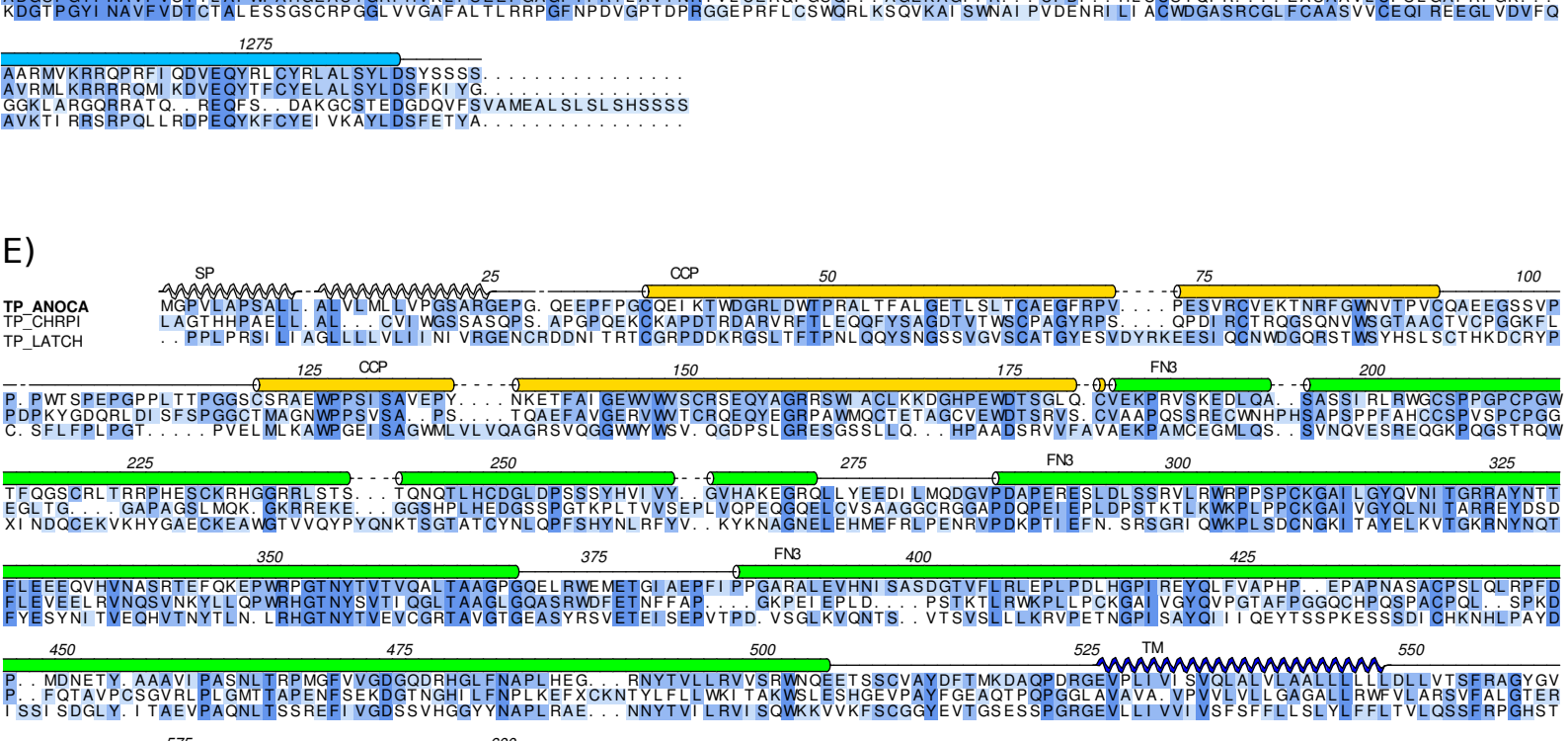

Supplement: S3 Fig — Amino acid similarity is indicated by a color saturation scale. The domain organization of the first sequence is shown on top as a reference; SP (gray): signal peptide, CCP (orange): complement control protein domain, FN3 (green): Fibronectin type III domain, PTP (light blue): protein tyrosine phosphatase domain, TM (blue): transmembrane region. Names combine Mystran (MYS), Trojan (TRO), Thracian (THR), Protein phosphatase (PP) or Transmembrane protein (TP) and the corresponding species abbreviation. Avian species: A. platyrhynchos (ANAPL), C. brachyrhynchos (CORBR), C. canorus (CUCCA), F. peregrinus (FALPE), F. albicollis (FICAL), G. fortis (GEOFO), M. gallopavo (MELGA), M. undulatus (MELUN), O. hoazin (OPPHO), T. guttata (TAEGU). Non-avian species: A. carolinensis (ANOCA), C. mydas (CHEMY), C. picta (CHRPI), L. chalumnae (LATCH). A) MSA of avian Mystrans; B) MSA of avian Trojans; C) MSA of avian Thracians; D) MSA of non-avian Protein phosphatases; E) MSA of non-avian Transmembrane proteins. (PDF) [file pone.0121672.s003.pdf]

A)

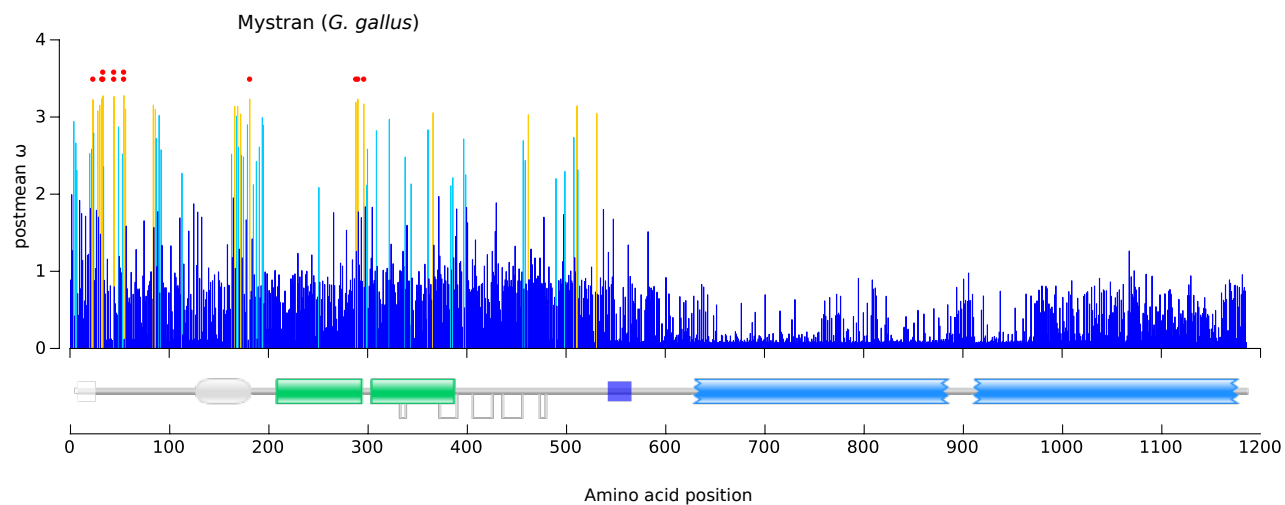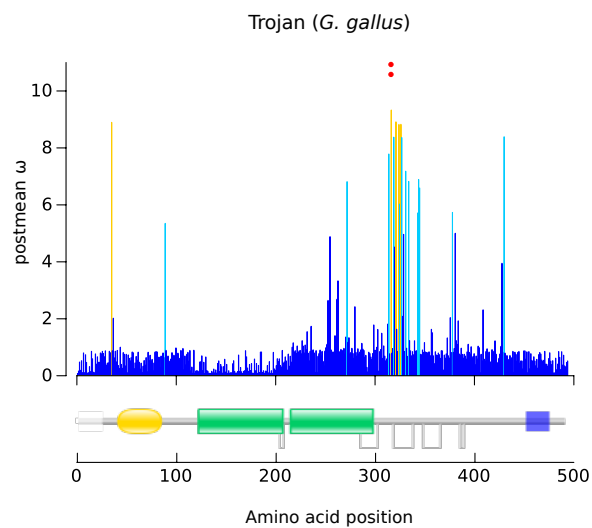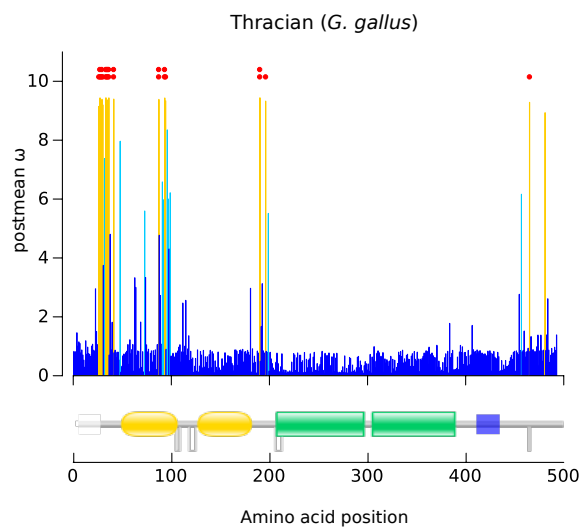

B)

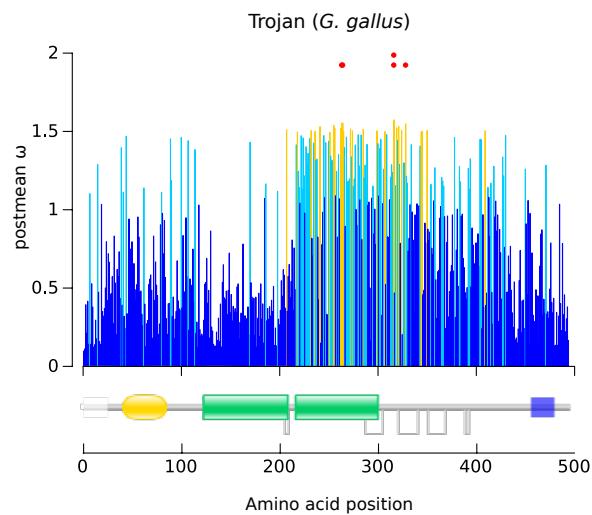

C)

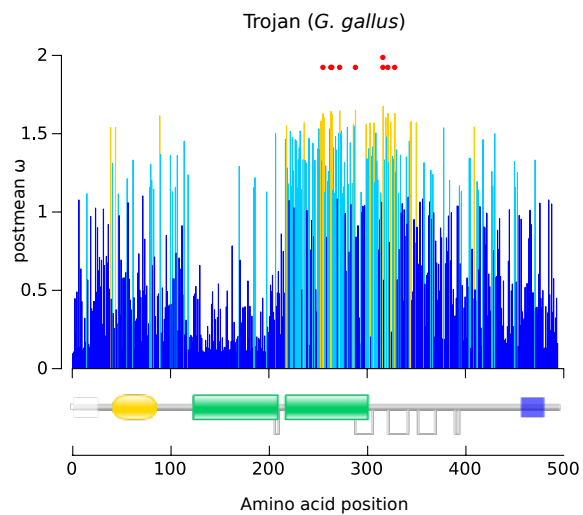

Signal peptide CCP domain FN3 domain PTP domain Transmembrane region ID region

Supplement: S4 Fig — Amino acid postmean ω values are mapped onto the protein topologies. Non selected sites are shown in blue, selected sites with probability below 90% are shown in light blue and selected sites with probability greater than 90% are shown in orange. Sites with probability greater than 95% and 99% are indicated by one or two red dots, respectively. Domain types and other topology properties are shown in the legend. The Mystran CCP domain is shown in gray scale, as it was predicted slightly below threshold, but had the expected position. A) Mystran, Trojan and Thracian analyzed without the sequences that appeared too divergent (MYS_ANAPL, TRO_FALPE and THR_FICAL, respectively). B) Trojan analyzed with TRO2_CUCCA omitted. C) Trojan analyzed with TRO2_CUCCA and TRO_FALPE omitted. (PDF) [file pone.0121672.s004.pdf]
