# Supplementary material for: Characterization of the Avian Trojan Gene Family Reveals Contrasting Evolutionary Constraints
Source: PLoS One. 2015 Mar 24;10(3):e0121672. doi: 10.1371/journal.pone.0121672 (PMC4372362; doi:10.1371/journal.pone.0121672)
Supplement: S2 Fig — Amino acid sequence identity is shown as a percentage. Names combine Mystran (MYS), Trojan (TRO) or Thracian (THR) and the corresponding species abbreviation. Species: A. platyrhynchos (ANAPL), C. brachyrhynchos (CORBR), C. canorus (CUCCA), F. peregrinus (FALPE), F. albicollis (FICAL), G. fortis (GEOFO), M. gallopavo (MELGA), M. undulatus (MELUN), O. hoazin (OPPHO), T. guttata (TAEGU). A) Mystrans; B) Mystrans, excluding cytoplasmic tails; C) Trojans; D) Thracians; (PDF) [file pone.0121672.s002.pdf]

A)

	MYS_ANAPL	MYS_CORBR	MYS_CUCCA	MYS_FALPE	MYS_FICAL	MYS_GALGA	MYS_GEOFO	MYS_MELGA	MYS_MELUN	MYS_OPHHO	MYS_TAEGU
MYS_ANAPL	100%	54%	51%	61%	52%	57%	54%	41%	54%	54%	55%
MYS_CORBR	54%	100%	60%	66%	65%	59%	64%	45%	59%	61%	68%
MYS_CUCCA	51%	60%	100%	69%	64%	64%	60%	50%	63%	65%	66%
MYS_FALPE	61%	66%	69%	100%	76%	77%	66%	60%	76%	68%	78%
MYS_FICAL	52%	65%	64%	76%	100%	65%	69%	54%	64%	61%	77%
MYS_GALGA	57%	59%	64%	77%	65%	100%	61%	69%	63%	63%	68%
MYS_GEOFO	54%	64%	60%	66%	69%	61%	100%	46%	59%	62%	73%
MYS_MELGA	41%	45%	50%	60%	54%	69%	46%	100%	50%	49%	54%
MYS_MELUN	54%	59%	63%	76%	64%	63%	59%	50%	100%	61%	64%
MYS_OPHHO	54%	61%	65%	68%	61%	63%	62%	49%	61%	100%	64%
MYS_TAEGU	55%	68%	66%	78%	77%	68%	73%	54%	64%	64%	100%

B)

	MYS_ANAPL	MYS_CORBR	MYS_CUCCA	MYS_FALPE	MYS_FICAL	MYS_GALGA	MYS_GEOFO	MYS_MELGA	MYS_MELUN	MYS_OPHHO	MYS_TAEGU
MYS_ANAPL	100%	18%	18%	18%	21%	26%	12%	22%	19%	18%	23%
MYS_CORBR	18%	100%	32%	25%	33%	33%	11%	32%	29%	30%	36%
MYS_CUCCA	18%	32%	100%	37%	47%	46%	25%	45%	34%	40%	47%
MYS_FALPE	18%	25%	37%	100%	65%	65%	27%	64%	52%	22%	65%
MYS_FICAL	21%	33%	47%	65%	100%	52%	30%	54%	46%	36%	61%
MYS_GALGA	26%	33%	46%	65%	52%	100%	31%	72%	43%	40%	55%
MYS_GEOFO	12%	11%	25%	27%	30%	31%	100%	30%	16%	23%	34%
MYS_MELGA	22%	32%	45%	64%	54%	72%	30%	100%	43%	39%	53%
MYS_MELUN	19%	29%	34%	52%	46%	43%	16%	43%	100%	26%	43%
MYS_OPHHO	18%	30%	40%	22%	36%	40%	23%	39%	26%	100%	39%
MYS_TAEGU	23%	36%	47%	65%	61%	55%	34%	53%	43%	39%	100%

C)

	TRO_ANAPL	TRO_CORBR	TRO1_CUCCA	TRO2_CUCCA	TRO_FALPE	TRO_FICAL	TRO_GALGA	TRO_GEOFO	TRO_MELUN	TRO_OPHHO	TRO_TAEGU
TRO_ANAPL	100%	32%	52%	39%	29%	42%	62%	30%	36%	41%	37%
TRO_CORBR	32%	100%	42%	36%	34%	51%	43%	51%	42%	45%	61%
TRO1_CUCCA	52%	42%	100%	62%	40%	50%	65%	41%	48%	54%	47%
TRO2_CUCCA	39%	36%	62%	100%	30%	36%	50%	36%	44%	46%	39%
TRO_FALPE	29%	34%	40%	30%	100%	34%	40%	27%	28%	45%	36%
TRO_FICAL	42%	51%	50%	36%	34%	100%	51%	47%	41%	44%	58%
TRO_GALGA	62%	43%	65%	50%	40%	51%	100%	41%	49%	55%	48%
TRO_GEOFO	30%	51%	41%	36%	27%	47%	41%	100%	38%	39%	56%
TRO_MELUN	36%	42%	48%	44%	28%	41%	49%	38%	100%	44%	41%
TRO_OPHHO	41%	45%	54%	46%	45%	44%	55%	39%	44%	100%	48%
TRO_TAEGU	37%	61%	47%	39%	36%	58%	48%	56%	41%	48%	100%

D)

	THR_ANAPL	THR_CORBR	THR_CUCCA	THR_FALPE	THR_FICAL	THR_GALGA	THR_GEOFO	THR_MELUN	THR_OPHHO	THR_TAEGU
THR_ANAPL	100%	32%	44%	39%	26%	46%	43%	42%	44%	41%
THR_CORBR	32%	100%	48%	48%	23%	46%	53%	46%	55%	54%
THR_CUCCA	44%	48%	100%	54%	30%	59%	64%	48%	67%	66%
THR_FALPE	39%	48%	54%	100%	28%	47%	52%	40%	54%	53%
THR_FICAL	26%	23%	30%	28%	100%	31%	28%	28%	30%	29%
THR_GALGA	46%	46%	59%	47%	31%	100%	54%	48%	59%	54%
THR_GEOFO	43%	53%	64%	52%	28%	54%	100%	45%	62%	82%
THR_MELUN	42%	46%	48%	40%	28%	48%	45%	100%	47%	46%
THR_OPHHO	44%	55%	67%	54%	30%	59%	62%	47%	100%	64%
THR_TAEGU	41%	54%	66%	53%	29%	54%	82%	46%	64%	100%

Legend: 10-25% 25-50% 50-70% 70-90% >90%
