# Supplementary material for: Characterization of the Avian Trojan Gene Family Reveals Contrasting Evolutionary Constraints
Source: PLoS One. 2015 Mar 24;10(3):e0121672. doi: 10.1371/journal.pone.0121672 (PMC4372362; doi:10.1371/journal.pone.0121672)
Supplement: S1 Table — The gene converted fragments between sequence pairs (Sequence I and Sequence II) are given with respect to their unaligned offsets and lengths within each sequence. “BC KA P-values”: Bonferroni-corrected KA (BLAST-like P-values). Names combine Mystran (MYS), Trojan (TRO) or Thracian (THR) and the corresponding species abbreviation. Species: A. platyrhynchos (ANAPL), C. brachyrhynchos (CORBR), C. canorus (CUCCA), F. peregrinus (FALPE), F. albicollis (FICAL), G. fortis (GEOFO), M. gallopavo (MELGA), M. undulatus (MELUN), O. hoazin (OPPHO), T. guttata (TAEGU). (PDF) [file pone.0121672.s005.pdf]

Sequence I	Sequence II	BC KA P-value	Fragment in Sequence I	Fragment in Sequence II
TRO_ANAPL	THR_ANAPL	1.36e-27	361-625 (265)	607-871 (265)
TRO_ANAPL	THR_ANAPL	1.74e-02	118-153 (36)	370-405 (36)
MYS_CORBR	TRO_CORBR	2.32e-46	1145-1480 (336)	878-1291 (414)
TRO_CORBR	THR_CORBR	4.20e-14	367-443 (77)	550-626 (77)
MYS_CORBR	THR_CORBR	1.31e-12	883-971 (89)	838-926 (89)
TRO_CORBR	THR_CORBR	5.54e-09	498-600 (103)	681-783 (103)
MYS_CORBR	TRO_CORBR	5.58e-02	949-997 (49)	724-772 (49)
TRO1_CUCCA	TRO2_CUCCA	1.4E−24	503-931 (429)	512-940 (429)
MYS_CUCCA	TRO1_CUCCA	5.0E−17	987-1158 (172)	735-906 (172)
MYS_CUCCA	TRO1_CUCCA	2.6E−16	577-895 (319)	322-640 (319)
MYS_CUCCA	TRO2_CUCCA	6.2E−16	987-1158 (172)	744-915 (172)
TRO2_CUCCA	THR_CUCCA	1.2E−13	650-740 (91)	914-1004 (91)
TRO1_CUCCA	THR_CUCCA	8.5E−12	641-731 (91)	914-1004 (91)
MYS_CUCCA	TRO2_CUCCA	8.3E−08	604-895 (292)	358-649 (292)
TRO_FALPE	THR_FALPE	2,86E-034	112-327 (216)	130-345 (216)
TRO_FALPE	THR_FALPE	4,09E-002	1270-1307 (38)	1273-1310 (38)
TRO_FICAL	THR_FICAL	1.01E-048	1342-1475 (134)	1156-1289 (134)
MYS_FICAL	TRO_FICAL	2.93E-041	1213-1600 (388)	955-1342 (388)
MYS_GALGA	TRO_GALGA	7,05E-027	598-1486 (889)	355-1243 (889)
TRO_GALGA	THR_GALGA	1,18E-003	270-316 (47)	519-565 (47)
TRO_GALGA	THR_GALGA	8,51E-003	331-356 (26)	580-605 (26)
MYS_GALGA	THR_GALGA	8,70E-003	297-307 (11)	312-322 (11)
MYS_GEOFO	TRO_GEOFO	6,24E-023	141-822 (682)	345-1069 (725)
TRO_GEOFO	THR_GEOFO	7,14E-004	289-298 (10)	556-565 (10)
TRO_GEOFO	THR_GEOFO	2,42E-002	186-230 (45)	450-494 (45)
TRO_MELUN	THR_MELUN	3,30E-016	278-594 (317)	416-732 (317)
TRO_MELUN	THR_MELUN	4,39E-005	661-788 (128)	799-926 (128)
MYS_MELUN	THR_MELUN	1,38E-003	475-482 (8)	373-380 (8)
MYS_MELUN	THR_MELUN	9,63E-003	498-515 (18)	396-413 (18)
MYS_OPHHO	TRO_OPHHO	1,39E-011	922-1006 (85)	749-833 (85)
MYS_OPHHO	TRO_OPHHO	2,70E-010	614-838 (225)	371-595 (225)
TRO_OPHHO	THR_OPHHO	2,62E-002	269-314 (46)	533-578 (46)
MYS_OPHHO	THR_OPHHO	6,61E-002	376-390 (15)	397-411 (15)
MYS_TAEGU	TRO_TAEGU	2,51E-020	552-845 (294)	300-593 (294)
MYS_TAEGU	THR_TAEGU	6,03E-007	895-1004 (110)	898-1007 (110)
MYS_TAEGU	TRO_TAEGU	2,80E-004	1454-1529 (76)	1055-1130 (76)
