# Supplementary material for: Characterization of the Avian Trojan Gene Family Reveals Contrasting Evolutionary Constraints
Source: PLoS One. 2015 Mar 24;10(3):e0121672. doi: 10.1371/journal.pone.0121672 (PMC4372362; doi:10.1371/journal.pone.0121672)
Supplement: S2 Table — Amino acids from chicken Mystran, Trojan and Thracian with Bayesian posterior probabilities to belong to site-class under positive selection are listed. Probability: >90%, >95% (*) or >99%(**), as inferred by Bayes-Empirical-Bayes (BEB). (PDF) [file pone.0121672.s006.pdf]

Gene	Omitted sequences	LL test (M8A vs. M8)	Sites with probability > 90%
Mystran	*MYS_ANAPL*	2ΔL = 96.9<br>P-value = 7.2E−23<br>ω = 2.6 (10%)	23H*, 28G, 30Y, 32G*, 33Y**, 44D**, 54T**, 56A, 84G, 86D, 166K, 169L, 172D, 181Q*, 288S*, 290R*, 296A*, 366T, 462P, 511S, 531I
Trojan	*TRO_FALPE*	2ΔL = 35.4<br>P-value = 2.7E−09<br>ω = 45.1 (6.2%)	35Q, 316G**, 321C, 324L, 326L
Thracian	*THR_FICAL*	2ΔL = 57.1<br>P-value = 4.2E−14<br>ω = 11.9 (6.4%)	26G*, 27A*, 28G*, 29A*, 30V*, 33K*, 34T*, 35E*, 36E*, 41E*, 87K*, 93G*, 94L*, 190T*, 196A*, 465S*, 481A
Trojan	*TRO2_CUCCA*	2ΔL = 23.7<br>P-value = 1.1E−06<br>ω = 1.6 (31.7%)	207S, 218Q, 232L, 236A, 241R, 251I, 255A, 256S, 262L, 263H*, 264M*, 265E, 272Q, 274T, 285T, 299P, 307S, 316G**, 319H, 321C, 324L, 328T*, 344Q, 345P, 350S, 409F
Trojan	*TRO_FALPE, TRO2_CUCCA*	2ΔL = 26.6<br>P-value = 2.5E−07<br>ω = 1.8 (26%)	39T, 44Q, 89V, 218Q, 236A, 253A, 255A*, 256S, 263H*, 264M*, 272Q*, 286R, 288V*, 299P, 303S, 307S, 316G**, 319H, 321C*, 324, 328T*, 344Q, 345P, 350S, 409F
